# Antibacterial PDT nanoplatform capable of releasing therapeutic gas for synergistic and enhanced treatment against deep infections

**DOI:** 10.7150/thno.70277

**Published:** 2022-02-28

**Authors:** Bingshuai Zhou, Xiaolin Sun, Biao Dong, Siyao Yu, Liang Cheng, Songtao Hu, Wei Liu, Lin Xu, Xue Bai, Lin Wang, Hongwei Song

**Affiliations:** 1 State Key Laboratory on Integrated Optoelectronics, College of Electronic Science and Engineering, Jilin University, Changchun, 130012, China; 2 Department of Oral Implantology, Jilin Provincial Key Laboratory of Sciences and Technology for Stomatology Nanoengineering, Hospital of Stomatology, Jilin University, Changchun, 130021, China

**Keywords:** antibacterial photodynamic therapy, anti-inflammation, carbon monoxide, reactive oxygen species, bacterial infections

## Abstract

Antibacterial photodynamic therapy (aPDT) has emerged as an attractive treatment option for efficient removal of pathogenic bacteria. However, aPDT in deep tissue will encounter difficulties such as limited light penetration depth, insufficient oxygen (O_2_) supply and inability to eliminate inflammation introduced by bacteria, which hinders its clinical application. Herein, the near infrared (NIR) strategy of simultaneously generating O_2_ and CO was developed for aPDT based antibacterial therapy and mitigation of deep infection inflammation.

**Methods:** We prepared NIR-mediated multifunctional aPDT nanoplatform (POS-UCNPs/ICG) producing therapeutic gas of O_2_ and CO. The CO, O_2_ and ROS generation of the nanoplatform were characterized by dye probes, respectively. The antibacterial activity and anti-inflammation of POS-UCNPs/ICG were demonstrated *in vitro* and* in vivo*. In addition, the therapeutic effects *in vivo* were serially analyzed by immunofluorescence staining, Masson's staining, hematoxylin and eosin staining, colony formation units (CFU) and so on.

**Results:** NIR-mediated multifunctional aPDT nanoplatform was realized by combining the up-conversion nanoparticles (UCNPs) and partially oxidized SnS_2_ (POS) nanosheets (NSs) as well as indocyanine green (ICG). Using a single 808 nm light, aPDT can be achieved via ICG molecules, meanwhile, O_2_/CO can be generated by POS NSs through upconversion light excitation. During the aPDT process, O_2_ can enhance aPDT, while CO can regulate inflammation through the PI3K/NF-κB pathway. Therefore, POS-UCNPs/ICG groups had a highest percentage of healing area up to 91.55±1.26% in mouse abscess model.

**Conclusion:** Due to enhanced aPDT and anti-inflammatory collaborative therapy, the POS-UCNPs/ICG composites showed remarkably accelerated recovery in animal abscess models. Such NIR light responsive nanoplatform with optimized antibacterial capacity and immunomodulatory functions is promising for clinical therapeutics of bacteria-induced infections.

## Introduction

Infection diseases induced by bacteria are a serious threat to human health with approximately 700,000 deaths worldwide every year 1. Pathogenic bacteria manipulate the host response and destructive inflammation, thereby leading to tissue destruction, such as abscess, periodontal disease, endophthalmitis, bacteremia and delaying wound healing 2-6. Antibiotics have been widely used for bacterial infections as the most accepted way of therapeutics. However, their improper application and overuse have led to the production of drug-resistant, eventually resulting in the refractoriness of the diseases.

As a promising approach, antibacterial photodynamic therapy (aPDT) has attracted increasing attentions for antibacterial applications, since it explores new routes to resolve the problem posed by major invasion of surgery and antibiotic resistance 7-9. In the course of aPDT, photosensitizers (PSs) with light irradiation can produce instantaneously and abundantly reactive oxygen species (ROS), which can destroy bacterial cell membrane and DNA, and then induce bacterial necrosis and apoptosis 10, 11. Many PSs including porphyrin 12-14, phthalocyanine 15, bodipy dyes 16, methylene blue 17, and rose bengal 18, *etc.*, can be triggered by ultra-violet (UV) or visible light. While considering the tissue depth of infectious diseases, the stimulation mode of PSs needs to be improved, since the penetration of UV and visible light is limited in tissue. Near infrared (NIR) light has longer penetration depth and better biosafety with minimal background interference, hence NIR- responsive PSs have been attracted great attention, such as typical NIR PS indocyanine green (ICG), which can produce substantial ROS upon 808 nm NIR light irradiation, becoming an important candidate for NIR based antibacterial treatments.

Although aPDT has made great progress, there are still two difficulties that need to be addressed before moving to clinical applications. First, according to the mechanism of aPDT, when oxygen is present, PSs can produce plentiful singlet oxygen under appropriate light irradiation. While the bacterial infections in deep sites possessed a hypoxic microenvironment, imposing restrictions on the oxygen-dependent aPDT 19. Second, aPDT only kills bacteria, but does not eliminate bacteria induced inflammation which damages the normal tissue 20. Therefore, novel aPDT strategies with oxygen self-supplication and inflammation elimination are highly desired.

In the internal environment, the nanomaterials to produce gas molecules by using environmental factors at the location of the lesion should be an effective solution to mitigate hypoxia and modulate local inflammation. Recent years, many studies have demonstrated that the oxygen-self-supplied aPDT nanoplatforms are an important method for solving hypoxia problem by using hydrogen peroxide (H_2_O_2_) in the body 15,21-24. Bu *et al*. developed the H_2_O_2_-responsive MnO_2_ nanosheets with up-conversion nanoprobe to solve hypoxic problems 25. Lin *et al.* designed an effective oxygen-self-supplied nanoplatform PEGylated CMS@GOx for PDT 26. In these works, nanozymes are introduced into the nanomaterial system to obtain oxygen. In our previous study, the oxygen self-supply MnO_2_ layers were introduced into NIR light triggered aPDT nanoplatform to solve the hypoxia problem in antibacterial treatment on the premise of H_2_O_2_ at infection sites 3. In these works, nanozymes were introduced into the nanomaterial system to obtain oxygen. In addition, photocatalytic material decorated nanoplatforms are also the way to provide oxygen 27.

For inflammation treatment, some gas molecules, such as NO, CO, H_2_S, which inhibit over-reacted inflammation via regulating the key signaling pathway 28-30, have shown the obvious anti-inflammatory properties 31-33. Among the gas molecules, colorless and odorless carbon monoxide (CO) shows important biological regulation at low concentrations and is widely used in anti-inflammation, organ transplantation, and cell metabolism modulation 32,34,35. Although the therapeutic effect of gas molecules was widely accepted, efficient gas molecule delivery in the lesion site is still restricted. When facing inflammation problems, aPDT capable of creating therapeutic gas molecules in the body deserves more efforts by designing composite structure with multiple functions. Moreover, accurately controlled CO release and delivery are of importance for the safe and efficient treatment of local inflammation 36,37.

**Figure 1:**
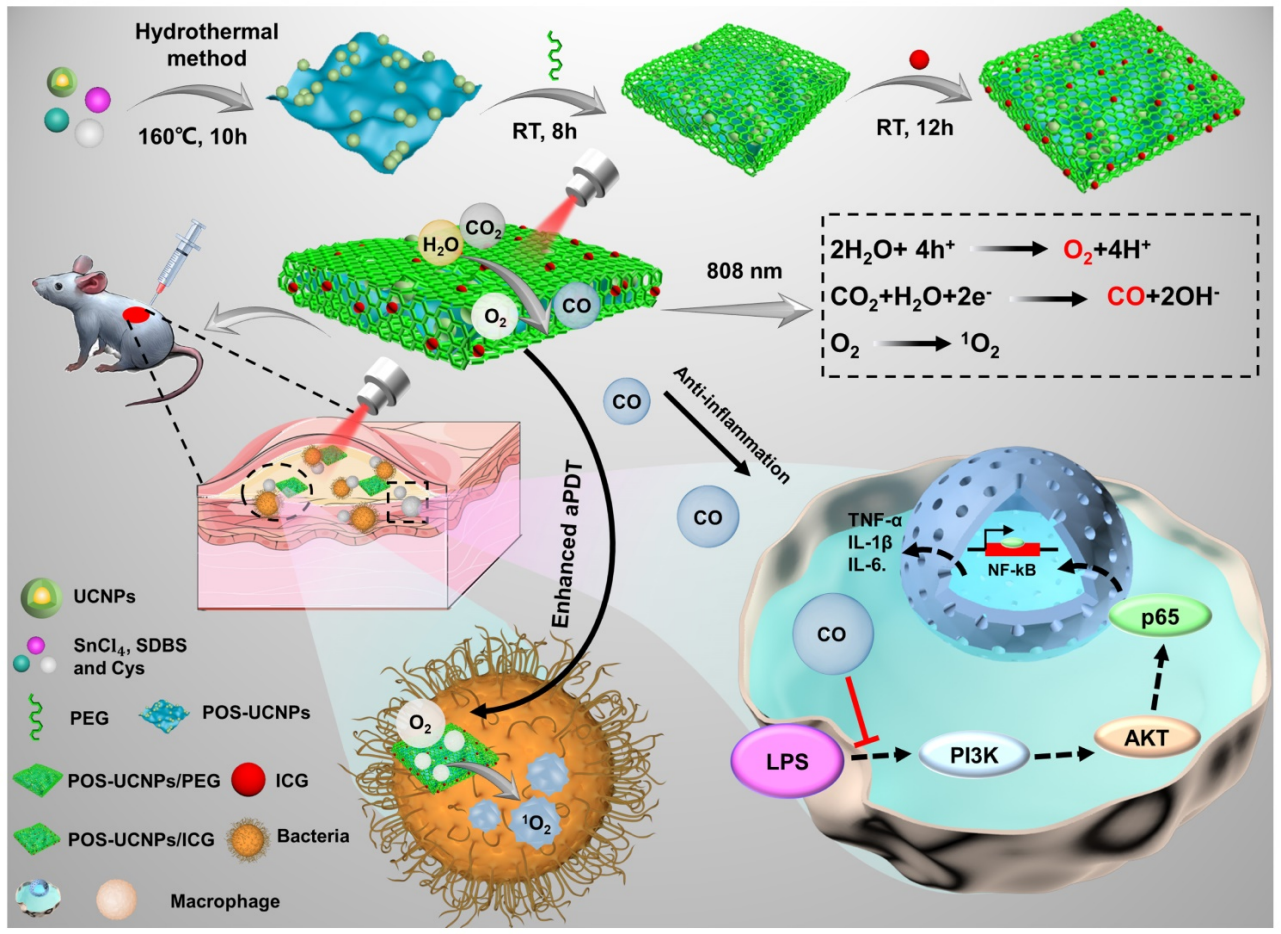
Schematic illustration of POS-UCNPs/ICG in preparation, antisepsis and anti-inflammatory mechanism. The antimicrobial property was due to the ROS produced by ICG under 808 nm light irradiation. POS-UCNPs/ICG could efficiently generate CO and O_2_ through up-conversion process of UCNPs with 808 nm excitation. The CO molecules could inhibit the inflammatory responses caused by lipopolysaccharide (LPS) stimulation through regulating the PI3K/NF-**κ**B signal pathway, and the O_2_ could be used to enhance the aPDT.

In this work, hypoxia and inflammation were considered at the same time by producing O_2_ and CO within a NIR mediated aPDT nanoplatform, which is composed of two-dimension (2D) partially oxidized tin disulfide (SnS_2_) nanosheets (POS NSs), UCNPs and ICG molecules (POS-UCNPs/ICG) as shown in Figure 1. The 2D POS NSs have the advantage of the high CO and O_2_ yield with visible light irradiation due to the high charge separation rate and large charge separation lifetime 38. While UCNPs can transform NIR light (808 nm) to green light for producing CO and O_2_ with POS NSs via reduction of CO_2_ and oxidization of H_2_O 39-42, sharing the same NIR excitation light with ICG molecules. Therefore, with 808 nm light only, ICG can produce large amounts of ROS with the O_2_ supplication. Simultaneously, the produced CO from the same up-conversion excitation process can efficiently inhibit the inflammatory responses through adjusting the PI3K/NF-**κ**B signal pathway. In animal models, this anti-inflammatory and enhanced antimicrobial activity lead to faster recovery of abscess sites. This nanoplatform provides a promising approach to solving hypoxia and the following inflammation treatment issues in aPDT, showing important potential in aPDT clinical applications.

## Materials and methods

### Materials

Sodium dodecyl benzenesulfonate (SDBS), L-cysteine (Cys), SnCl_4_·5H_2_O and ethanediol were provided by Shanghai Aladdin Biochemical Technology Co., Ltd. Hexadecyl trimethyl ammonium Bromide (CTAB), cyclohexane, oleic acid (OA) and 1-Octadecene (ODE) were purchased from Macklin. Ethanol was purchased from Beijing Chemical Works. Yttrium (III) chloride hexahydrate (YCl_3_•6H_2_O), ytterbium chloride hexahydrate (YbCl_3_•6H_2_O), erbium chloride hexahydrate (ErCl_3_•6H_2_O) and neodymium chloride hexahydrate (NdCl_3_•6H_2_O) were provided by Ruike Centre. Cell Counting Kit-8 (CCK-8) were obtained from Beijing Solarbio. 2',7'- dichlorodihydrofluorescein diacetate (DCFH-DA) was purchased from Sigma. All chemicals were directly used without further purification.

### Preparation of NaYF_4_:20%Yb^3+^,2%Er^3+^@NaYF_4_:20%Yb^3+^,30%Nd^3+^ core-shell NPs

OA-coated NaYF_4_:Yb^3+^,Er^3+^ NPs were traditionally synthesized by solvothermal method. Firstly, 0.78 mmol YCl_3_•6H_2_O, 0.2 mmol YbCl_3_•6H_2_Oand 0.02 mmol ErCl_3_•6H_2_O were put into 100 mL three-necked flask. Then 6 mL OA and 15 mL ODE were put into the flask. Next the mixed solution was heated to 130 °C for 60 min until the particles were completely dissolved, then the mixed solution was cooled down to ~ 35 °C. 4 mmol NH_4_F and 2.5 mmol NaOH were dissolved with 5-6 mL methyl alcohol under the condition of ultrasound and the resulting solution was added dropwise to the flask stirring for 30 min. The mixed solution was heated to 125 °C to remove the methyl alcohol. After that, the mixed solution was heated to 320 °C for 90 min. The whole process was protected by nitrogen. The mixture was cooled down to 35 °C and was collected by centrifugation at 9500 rpm for 15 min. The resulting sediments were washed with cyclohexane and absolute ethyl alcohol (v: v=1:2) and dispersed in cyclohexane for further modification and characterization.

The method of OA-coated NaYF_4_:Yb^3+^,Er^3+^@NaYF_4_:Yb^3+^,Nd^3+^ NPs was the same as that of the core. Firstly, YCl_3_•6H_2_O (0.25 mmol), YbCl_3_•6H_2_O (0.1 mmol) and NdCl_3_•6H_2_O (0.15 mmol) were put into the three-necked flask with 6 mL OA and 15 mL ODE. Next the mixed solution was heated to 130 °C for 1 h until the particles were fully dissolved, then the mixed solution was cooled down to 35 °C. 6 mL cyclohexane containing 1 mmol NaYF_4_:Yb^3+^,Er^3+^ NPs then was added to the flask. After persistently stirred for 0.5 h, the solution was heated to 90 °C until the bubbles disappear, and the solution then was cooled naturally again to 40 °C. The subsequent steps were consistent with the core process, except that the time at 320 ℃ was 60 min.

### Preparation of POS-UCNPs/ICG nanosheets

POS-UCNPs nanosheets (NSs) were synthesized according to the previous work 39. Briefly, 0.418g SDBS, 0.1754g SnCl_4_·5H_2_O, 0.4846g Cys and CTAB modified UCNPs were dispersed in 60 mL deionized (DI) water and ethylene glycol mixed solution (v: v =1:1). After fully stirred for 2 h, the mixed solution was put into 20 mL Teflon-lined autoclave and held at 160 °C for 10 h. The sediments were collected by centrifugation, and were washed with DI water and ethanol 3 times. Finally, POS-UCNPs NSs were stored at 4 °C.

10 mg POS-UCNPs NSs were suspended in 30 mL DI water with ultrasound and ice bath conditions for 4 h. Next, the solution was centrifuged at 3000 rpm removing particles with large size, and the suspension liquid with POS-UCNPs NSs was collected by centrifugation at 9000 rpm for 5 min. The resulting sediments were suspended in DI water for further modification and characterization.

The 1 mg·mL^-1^ POS-UCNPs NSs and SH-PEG (m: m=5:1) were placed in glass bottles containing 10 mL DI water. Then ultrasound for 10 min, the products were persistently stirred for 8 h at room temperature (RT). The resulting products were centrifuged and washed with DI water for three times.

The resulting mixture (1 mg·mL^-1^) and the ICG solution (1 mg·mL^-1^) were mixed at a mass ratio of 10:1. The mixtures were stirred at room temperature for 12 h after 10 min of ultrasound. The resulting sediments were centrifuged and washed with DI water for three times.

### Characterization

The morphology images of nanomaterials were recorded on the Hitachi H-8100IV transmission electron microscopy (TEM) and scanning electron microscopy (SEM). The absorption wavelengths were tested by Shimadzu UV-3600. The size of the as-prepared samples were characterized by Zetasizer Nano-ZS ZEN3700 dynamic light scattering (DLS). The phase structure of UCNPs and POS NSs were measured by RigakuTTR III X-ray power diffractometer (XRD). The cytotoxicity of sample was analyzed by microplate reader (Bio-Tek ELX800, USA). The Fourier transform infrared (FTIR) spectroscopy of samples were characterized by Shimadzu Fourier infrared spectrometer.

### Cytotoxicity assay

L-929 cells were seeded at a density of 1×10^4^ cells/well in 96-well plates for 24 h and then co-cultured with different concentrations of POS-UCNPs/ICG for another 24 h. Subsequently, CCK-8 assay was used to estimate the cell viability by micro-plate reader.

### Immunomodulatory activities of POS-UCNPs/ICG *in vitro*

#### Suppression of LPS induced PI3K and NF-κB activation in macrophages

The Raw 264.7 macrophages were seeded at a density of 2.5×10^5^ per well into a 6-well plate for 24 h. Firstly, the cells were stimulated with 2 μg·mL^-1^ *E. coli*-LPS for 3 h to mimic the inflammatory environment *in vitro*. Afterwards, the cells were co-incubated with PBS, POS-UCNPs, POS/ICG and POS-UCNPs/ICG (100 μg·mL^-1^) for 4 h, followed by 808 nm light irradiation (1 W·cm^-2^, 5 min) 39. After incubation at 37 °C for another 3 h, the cells were washed with PBS and fixed with 4% paraformaldehyde, followed overnight incubation at 4 °C with mouse anti-PI3K p110β primary antibodies (1:300, Santa Cruz). Subsequently, the cells were cultured with the Alexa Fluor 673-labeled goat anti-mouse IgG secondary antibodies (1:500, Beyotime) for 1 h at RT. Then, the cell nucleus was stained with 4, 6-diamidino-2-phenylindole (DAPI, Sigma) for 15 min in dark condition. Eventually, the macrophages were imaged by CLSM.

The steps of detecting NF-**κ**B activity were the same as those of detecting PI3K activity, except that the macrophages were incubated overnight at 4 °C with primary antibodies (rabbit anti-NF-**κ**B p65, 1:500, Bioss), following which the cells were cultured with the Alexa Fluor 488-labeled goat anti-rabbit IgG secondary antibodies (1:500, Beyotime) for 1 h at RT.

#### Cell cytokines detection

As above mentioned,* E. coli*-LPS activated Raw 264.7 macrophages were co-incubated with various nanomaterials with or without 808 nm light. After incubation for another 3 h, the macrophages were evaluated by the mRNA expression level of M1 markers (tumor necrosis factor-α (TNF-α), chemokine interleukin-6 (IL-6) and interleukin-1β (IL-1β)) and M2 markers (arginine-1 (Arg-1), transforming growth factor-β (TGF-β) and chemokine interleukin-10 (IL-10)) by qPCR method. The housekeeping gene β-actin as the reference gene was used to standardize the data. The qPCR values of blank control group, the cells without LPS stimulation, were calibrated as “1”.

### Antibacterial properties of POS-UCNPs/ICG

#### Biofilm Formation

*Staphylococcus aureus* (*S. aureus*, ATCC 29213) and *Escherichia coli* (*E. coli*, ATCC 25 922) were selected for biofilm formation and cultured in Tryptone Soy Broth (TSB) medium. For anti-biofilm activity, biofilm formation was performed as previous studies 3. Briefly, the sterilized glass slides were covered with 100 μL of bacterial suspension (10^7^ CFU·mL^-1^). Afterwards, 900 μL TSB medium containing various nanomaterials were respectively added into the bacterial suspension in the 24-well plate. The medium was changed for every day. 4 days later, mature biofilms were formed. The mature biofilms were then divided into four groups:

(1) Control groups: the mature biofilms were treated by PBS solution with or without 808 nm (1 W·cm^-2^, 5 min per day);

(2) POS-UCNPs groups: the mature biofilms were treated by 91.59 μg·mL^-1^ POS-UCNPs solution with or without 808 nm (1 W·cm^-2^, 5 min per day);

(3) POS/ICG groups: the mature biofilms were treated by POS/ICG (7.41 μg·mL^-1^ ICG) solution with or without 808 nm (1 W·cm^-2^, 5 min per day);

(4) POS-UCNPs/ICG groups: the mature biofilms were treated by 100 μg·mL^-1^ POS-UCNPs/ICG solution with or without 808 nm (1 W·cm^-2^, 5 min per day).

#### Antibacterial tests

For dead/live bacterial analysis, *S. aureus* and *E. coli* biofilms were respectively stained with a mixture of 2.5 μM SYTO9 and 2.5 μM propidium iodide (Live/Dead Baclight Bacterial Kit). Subsequently, the biofilms were imaged using CLSM.

For biofilm CFU count analysis, the biofilms were then collected from the specimen by mechanical scraping and ultrasound. The suspension of the biofilm was diluted 10 times at a time and streaked onto over the Luria-Bertani (LB) agar. The inverted agar plate was incubated at 37℃ and 5% CO_2_ for 24 h, and then CFU was calculated.

For bacterial SEM images, *S. aureus* and *E. coli* were respectively cultured with various nanomaterials at 24-wells plate for 24 h. Then bacteria were irradiated with 808 nm for 5 min. After various treatments, bacterial samples were collected and centrifuged (4000 rpm, 3 min). Afterwards, the bacterium was immobilized using 2.5% glutaraldehyde for 12 h. Then, the bacterium was dehydrated with different proportions of ethanol solution. Finally, the samples were observed using SEM.

### Therapeutic effect of POS-UCNPs/ICG against bacteria induced infection* in vivo*

#### Animal model

All BALB/c male mice used in the experiment were purchased from Liaoning Changsheng Biotechnology Co. Ltd. All animal experiments were performed according to the guidelines of the Institutional Animal Care and Use Committee. The abscess model was established according to the agreement authorized by Jilin University (JLUKQ #SY202105013). To establish the model of subcutaneous abscess, 100 μL* S. aureus* (10^8^ CFU·mL^-1^) were injected subcutaneously into the back of rat that had been shaved and sterilized. The subcutaneous abscess model mice were divided into four groups (five mice per group): each group was injected with 200 μL (1) Saline; (2) POS-UCNPs (91.59 μg·mL^-1^); (3) POS/ICG (7.41 μg·mL^-1^ ICG); (4) POS-UCNPs/ICG (100 μg·mL^-1^). 5 min after bacteria injection, saline, POS-UCNPs, POS/ICG, and POS-UCNPs/ICG were subcutaneously injected into the backs treated with bacteria, respectively. Next, all of those mice were irradiated with 808 nm light (1 W·cm^-2^, 5 min). The infected sites were observed on days 1, 5, 9, and 14 to observe the therapeutic effect of various nanomaterials. The size of the infected site was calculated by the Image J software. For evaluating antibacterial activity, viable counts of infected sites were carried out by the standard plate count method on 5 days. After 14 days, the mice were euthanatized, and these infected tissues were collected for histomorphometry analysis, immunofluorescence analysis and qPCR.

#### Histomorphometric and immunofluorescence (IF) analyses

The infected tissues were collected and fixed with 4% paraformaldehyde for 24 h. Subsequently, the infected tissues were embedded, sectioned, and stained with hematoxylin and eosin (H&E) and Masson staining.

Immunofluorescence analysis was used to detect M1 biomarker (TNF-α) and M2 biomarker (TGF-β) in collected tissues. Rabbit anti-TNF-α and rabbit anti-TGF-β as primary antibodies were co-incubated with samples, respectively. The primary antibodies were then removed, and biotinylated goat anti-rabbit as secondary antibodies were co-incubated with the samples for 60 min under dark condition. The samples then were treated with a solution of DAPI. The final samples were imaged using CLSM.

### Statistical analysis

All statistical analyses were evaluated using SPSS (19.0) software (SPSS, Chicago, IL, USA). The data were expressed in terms of means and standard deviation and were analyzed statistical significance by One-way analysis of variance (ANOVA). The mean of each group with p value of 0.05 was tested by Tukey multiple comparison.

## Results and Discussion

### The synthesis and characterization of POS-UCNPs/ICG

As shown in the schematic (Figure 1), POS-UCNPs/ICG with a single 808 nm NIR light irradiation achieved enhanced sterilization and anti-inflammatory functions though generating O_2_ and CO, which is the feature of this work. 808 nm NIR light has the largest tissue penetration depth in first NIR window 43. Based on the composite POS-UCNPs/ICG, the up-converted green light could be generated by UCNPs with 808 nm excitation, which served as the visible light source for photocatalysis of POS NSs. With the same excitation light, the aPDT function from ICG molecules was also obtained. O_2_ and CO gases produced from POS NSs played an important role for enhancing aPDT and relieving inflammation. UCNPs had a core-shell structure. The outer NaYF_4_:Yb^3+^,Nd^3+^ shell not only protected the inner core from the surface defects quenching, but also played a role in energy transfer. The harvested energy could be transferred from Nd^3+^ ions to the Yb^3+^ ions in the shell layer, which then sensitized the Yb^3+^ ions in the core, or Nd^3+^ ions transferred their energy to the Yb^3+^ ions in the core directly 44. The POS-UCNPs NSs composites prepared by hydrothermal method 45 were further modified with thiol-polyethylene glycol (SH-PEG), achieving hydrophilicity and biocompatibility. After ICG loading into the composite, the enhanced aPDT as well as the inflammatory regulation could be realized simultaneously.

**Figure 2:**
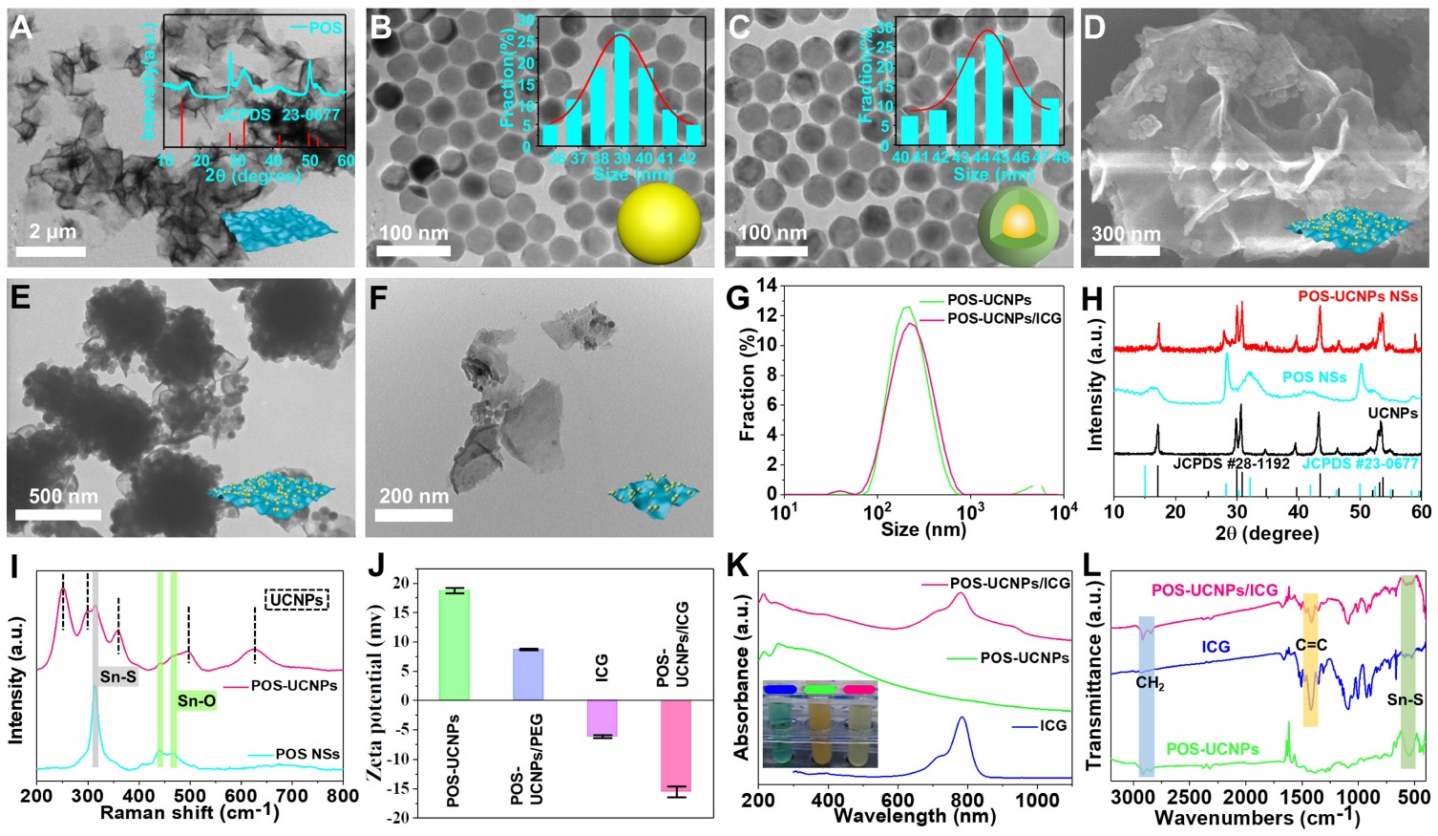
Characterization of POS-UCNPs/ICG. TEM images of (A) POS NSs, (B) NaYF_4_:Yb^3+^,Er^3+^ and (C) NaYF_4_:Yb^3+^,Er^3+^@NaYF_4_:Yb^3+^,Nd^3+^. The inset of A: XRD of POS NSs. The inset of B-C: the histograms of the corresponding particle size distribution. (D) SEM and (E) TEM images of POS-UCNPs NSs. (F) TEM image of POS-UCNPs after ultrasound. (G) The size distribution of POS-UCNPs and POS-UCNPs/ICG by DLS. (H) XRD of UCNPs, POS NSs and POS-UCNPs. (I) Raman spectra of POS NSs and POS-UCNPs. (J) ζ-potential of POS-UCNPs, POS-UCNPs/PEG, ICG and POS-UCNPs/ICG. (K) UV-vis-NIR absorption spectra of POS-UCNPs, ICG, and POS-UCNPs/ICG. The photos in the illustration are ICG (74 μg·mL^-1^), POS-UCNPs NSs (1 mg·mL^-1^) and POS-UCNPs/ICG (1 mg·mL^-1^). (L) FTIR spectra of POS-UCNPs, ICG and POS-UCNPs/ICG.

TEM (Figure 2A) and SEM (Figure S1) images showed that the prepared POS sample was flaky. The XRD pattern of POS NSs in Figure 2A could be attributed to hexagonal SnS_2_ standard card (JCPDS No.23-0677). Moreover, Raman spectroscopy demonstrated that Sn was partially oxidized due to the appearance of Sn-O characteristic peaks at 438 cm^-1^ and 466 cm^-1^ (Figure S2). The content of Sn oxide could be calculated quantitatively by XPS to be 3.3% (Figure S3), which was comparable to the previous literature with the highest CO generation rate 45. Morphology and size of NaYF_4_:Yb^3+^,Er^3+^ and NaYF_4_:Yb^3+^,Er^3+^@NaYF_4_:Yb^3+^,Nd^3+^ were shown in Figure 2B-C. After the shell formation of NaYF_4_:Yb^3+^,Nd^3+^, the size of the nucleus increased from 38.95±3.3 nm to 44.7±4.0 nm, while the morphology of UCNPs was almost unchanged. The homogeneous coating of NaYF_4_:Yb^3+^,Nd^3+^ shell modification was further confirmed by XRD patterns of the inner core and core/shell structure (Figure S4).

Figure 2D-E showed the composites combined UCNPs and POS NSs, and POS-UCNPs NSs with the average thickness of 7 nm (Figure S5). POS-UCNPs NSs with large size were shattered into small fragments by ultrasound as shown in Figure 2F and Ultrasound did not affect the performance of POS-UCNPs NSs to produce O_2_ and CO (Figure S6). The corresponding DLS analysis showed that the diameter of POS-UCNPs was about 200 nm (Figure 2G). The XRD pattern of POS-UCNPs displayed the characteristic peak of POS NSs as well as UCNPs (Figure 2H). From the XPS spectrum of POS-UCNPs, S, Sn and O elements, which constituted the POS NSs, could be observed (Figure S7A), and Yb, Er, Nd and Y elements from UCNPs also appeared (Figure S7B-G). The characteristic Raman peaks of UCNPs appeared at 251 cm^-1^, 303 cm^-1^, 359 cm^-1^, 492 cm^-1^, 625 cm^-1^, and that of POS NSs at 317 cm^-1^ (Sn-S), 438 cm^-1^ and 466 cm^-1^ (Sn-O) further proved the successful preparation of the POS-UCNPs NSs (Figure 2I and Figure S8) 46, 47.

In order to improve the solubility and biocompatibility of POS-UCNPs, we modified POS-UCNPs with the polymer SH-PEG (POS-UCNPs/PEG). The photosensitizer ICG was loaded into POS-UCNPs/PEG composites via electrostatic adsorption, obtaining POS-UCNPs/ICG. The hydrodynamic size of the POS-UCNPs increased from 200 nm to 220 nm and the zeta potential reversed from 18.75 mV to -16.27 mV after coating polymer and ICG molecules (Figure 2G and 2J). In addition, absorption spectra also confirmed that all the ingredients were present in POS-UCNPs/ICG (Figure 2K). FTIR spectra in Figure 2L confirmed the existence of ICG and POS NSs. The C=C double bond of 1423 cm^-1^ in FTIR indicated the existence of ICG molecules. The Sn-S bond in FTIR at 540 cm^-1^ confirmed the presence of POS NSs. CH_2_ group in POS-UCNPs/ICG was significantly enhanced at 2854 cm^-1^ and 2924 cm^-1^ due to the addition of PEG. According to the absorption of ICG at 780 nm, the encapsulation rate of ICG onto POS-UCNPs/ICG was calculated to be 74.1 wt%. Dispersion performance of POS-UCNPs/ICG in deionized water, PBS and culture medium and long-term stability in deionized water were also studied (Figure S9-10). These results confirmed that POS-UCNPs/ICG had good dispersibility and long-term stability.

### Detection of CO, O_2_ and ROS *in vitro*

**Figure 3:**
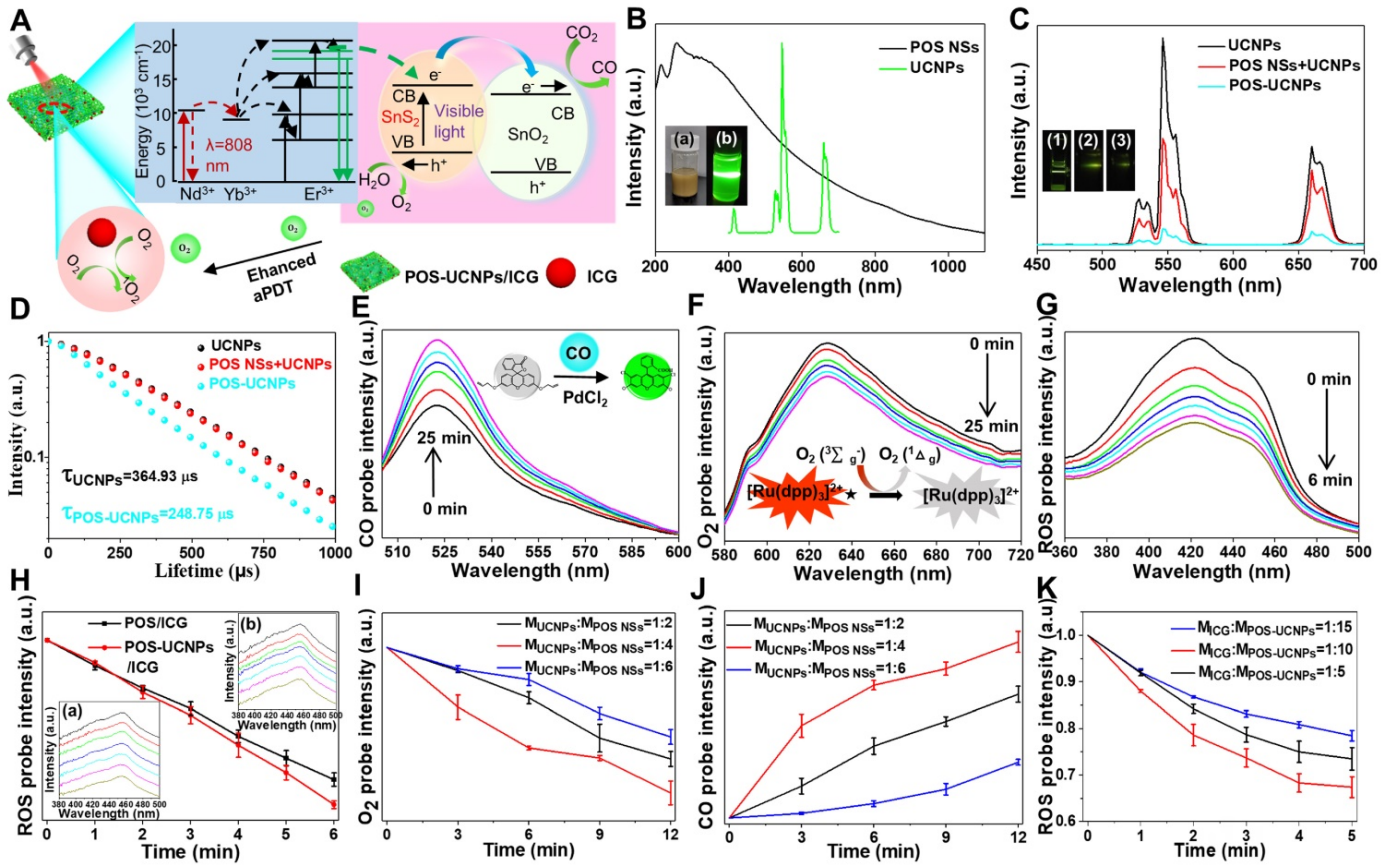
* In vitro* evaluation of CO, O_2_ and ROS generation rate of POS-UCNPs/ICG under 808 nm light irradiation. (A) Schematic diagram of O_2_, CO and ROS generation by POS-UCNPs/ICG. (B) The black line: the absorption spectrum of POS NSs. The green line: the emission spectrum of UCNPs. Insets: digital photo of a) POS NSs and that of b) UCNPs with 808 nm light irradiation. (C) Emission spectra of UCNPs, UCNPs+POS NSs and POS-UCNPs. Insets: digital photo of (1) UCNPs, (2) UCNPs+POS NSs and (3) POS-UCNPs with 808 nm light. (D) The lifetime of UCNPs, UCNPs+POS NSs and POS-UCNPs. Variation of fluorescence intensity of POS-UCNPs/ICG solution with (E) CO probe and (F) O_2_ probe. (G) Absorbance spectra at 420 nm of DPBF probe in the presence of POS-UCNPs/ICG. (H) The variation of ROS probe luminescent intensity containing POS/ICG and POS-UCNPs/ICG. Inset: The changes of ROS probe luminescent intensity containing a) POS-UCNPs/ICG and b) POS/ICG. (I) O_2_ and (J) CO production with different mass ratios of UCNPs: POS NSs. (K) ROS generation at different mass ration of ICG: POS-UCNPs.

For POS-UCNPs/ICG, it was important to explore ROS generating rate and the photocatalytic ability of POS NSs by upconversion triggering (Figure 3A). The CO and O_2_ generating capability of POS-UCNPs under 546 nm light was firstly detected by the corresponding probes. POS-UCNPs NSs could successfully catalyze the formation of O_2_ and CO (Figure S11-12) and UCNPs did not affect the photocatalytic performance of POS-UCNPs. The emission of UCNPs and the absorption of POS NSs were tested (Figure 3B), which supported the possibility of energy transfer. Considering that the energy transfer process was also affected by the distance between the donor and the receptor, the emission spectra of UCNPs, UCNPs and POS NSs mixed solution and POS-UCNPs NSs were tested. The results showed a successive decrease the emission intensity (Figure 3C), indicating the non-radiative energy transfer between POS NSs and UCNPs due to the close combination. To further verify the energy transfer mechanism, the 546 nm fluorescence lifetime of UCNPs, UCNPs and POS NSs mixture and POS-UCNPs NSs were tested. The lifetime of POS-UCNPs decreased from 364.93 μs (UCNPs) to 248.75 μs, while the fluorescence lifetime of the mixed solution of UCNPs and POS NSs did not change, as shown in Figure 3D.

Gas generation and aPDT capability of POS-UCNPs/ICG composites were studied by detecting CO, O_2_ and ^1^O_2_. Under the catalysis of PdCl_2_, CO probe could emit green fluorescence when CO was present 48. Under irradiation of 808 nm light, the fluorescence intensity of the solution at 522 nm continued to increase, which clearly demonstrated the continuous CO generation (Figure 3E). More importantly, POS-UCNPs/ICG could continue to produce CO even after 25 min under irradiation, providing sufficient CO for anti-inflammatory action at the cellular and animal levels. The O_2_ production capacity of POS-UCNPs/ICG was evaluated with Tris (4,7-diphenyl-1,10-phenanthroline) ruthenium (II) dichloride O_2_ probe. Figure 3F showed that the red-light intensity of the solution at 628 nm gradually decreased due to the O_2_ quenching, indicating efficient O_2_ support for aPDT.

ROS production capacity of POS-UCNPs/ICG composites was tested as shown in Figure 3G. The absorption intensity of ROS probe DPBF at 420 nm decreased rapidly after 808 nm light irradiation for 6 min, indicating the efficient ROS generation. To further evaluate the O_2_ self-supply function, the ROS production rate of POS/ICG and POS-UCNPs/ICG groups was compared by monitoring ROS probe fluorescence intensity. Figure 3H showed that the ROS production rate of both groups was nearly the same in the first three min, indicating that in the early stage of PDT, O_2_ was sufficient and the increased O_2_ had little effect on the ROS generation rate. While in the last 3 min, the O_2_ from POS-UCNPs/ICG caused more ROS production (Figure 3H). These results showed that when the treatment time exceeded 3 min, the self-supply of O_2_ would play an important role. Obviously, in this process, UCNPs were very important in the production of O_2_ because of their efficient up-conversion fluorescence.

What's more, this O_2_ self-supply function could be observed among the POS-UCNPs/ICG composites with different ratios of UCNPs/POS NSs, and the optimal ratio was also studied. Figure 3I revealed that the UCNPs: POS NSs of 1:4 showed a highest O_2_ yield under the same conditions. Since O_2_ and CO were produced simultaneously, the optimal ratio of UCNPs and POS NSs was also explored by CO production rate as shown in Figure 3J. Among the UCNPs/POS NSs samples, the mass ratio of 1:4 sample also showed the highest CO yield under the same conditions, which was similar to that of O_2_ generation tests. In addition, we also quantitatively detected the amount of O_2_ and CO which were ~ 0.75 μM and ~ 1.5 μM, respectively, under the same condition (Figure S13). In addition, we performed commonly used kinetic model fitting for CO and O_2_ from POS-UCNPs/ICG. The zero-order kinetic model coefficients (R^2^) of CO and O_2_ were ~ 0.98 and ~ 0.96, respectively, proving that the production of CO and O_2_ under NIR irradiation was more consistent with the zero-order kinetic model, proving that POS-UCNPs /ICG could stably produce CO and O_2_ at the condition of 1 W/cm^-2^ in a certain time (Figure S14).

To account for the 1:4 POS-UCNPs NSs producing the most O_2_ and CO, we examined the lifetime of UCNPs and POS-UCNPs NSs with different proportions (Figure S15). As the mass ratio of UCNPs decreases, the lifetime of 546 nm gradually decreased from 364.93 μs (UCNPs) to 333.93 μs (1:2 POS-UCNPs NSs), 248.75 μs (1:4 POS-UCNPs NSs), and 147.18 μs (1:6 POS-UCNPs NSs). This indicated that the energy transfer between UCNPs and POS NSs increased with the decrease of UCNPs mass ratio. However, when the mass was constant, a large proportion of UCNPs in 1:2 POS-UCNPs/ICG could generate more green light that POS NSs could absorb, but a small amount of POS NSs led to a decrease in the output of O_2_ and CO. The proportion of UCNPs in 1:6 POS-UCNPs/ICG was relatively small, resulting in a production decrease of the green light, which led to a decreased production of O_2_ and CO. Therefore, when UCNPs: POS NSs was 1:4 in POS-UCNPs/ICG, the energy utilization was maximum, and the production of O_2_ and CO was also the highest among the sample (Figure 3I-J). We analyzed XPS data of 1:4 POS-UCNPs NSs as shown in Table S2. The mass ratio of UCNPs to POS NSs was 1:2.8 when the feed ratio is at 1:4.

The ICG molecules were responsible for the production of ROS, and its maximum loading amount was studied. When the mass ratio of ICG to POS-UCNPs NSs was 1:10, the highest ICG molecule loading rate was realized (Figure S16), and therefore, more ROS could be generated (Figure 3K).

### Detection of intracellular CO, ROS and biosafety assay

The intracellular CO production capacity of POS-UCNPs/ICG upon 808 nm NIR irradiation was detected with the same CO probe* in vitro*. Figure S17A-B showed that intensive green fluorescence signal could be observed in the illuminated groups of POS-UCNPs and POS-UCNPs/ICG, which proved the remarkable CO production capacity of the nanoplatform in the presence of POS-UCNPs NSs. However, in other groups, there was only weak green fluorescence signal from CO probe. In addition, DCFH-DA was applied to investigate the ROS generation at the cellular level. As shown in Figure S17C, the cells in the POS/ICG group and POS-UCNPs/ICG group with 808 nm light irradiation showed an intensive green fluorescence signal, indicating the favorable capacity of ROS generation. While, in other groups, the green fluorescence was very weak. Figure S17D displayed the fluorescence intensity of POS/ICG and POS-UCNPs/ICG groups with NIR light irradiation was stronger than other groups. In particular, POS-UCNPs/ICG group exhibited the superior capacity of ROS generation, since the local O_2_ generation upon 808 nm NIR light irradiation happened.

**Figure 4:**
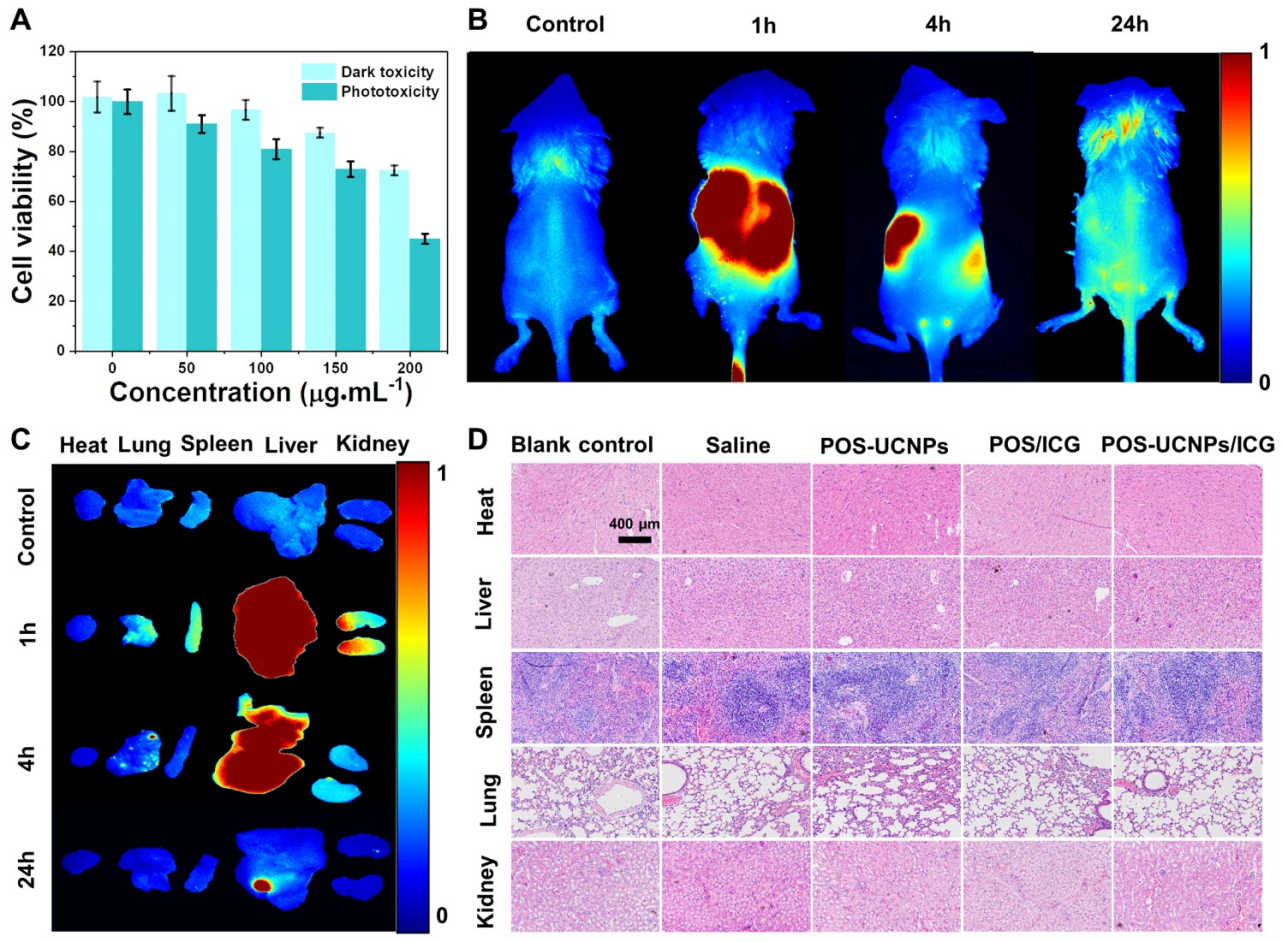
The biosafety assay of POS-UCNPs/ICG. (A) Dark toxicity and phototoxicity (1 W·cm^-2^, 5 min) of POS-UCNPs/ICG at different concentrations. (B) Control, 1 h, 4 h and 24 h animal imaging of POS-UCNPs/ICG metabolism in *vivo* (Control group was injected with PBS). (C) Control, 1 h, 4 h and 24 h organs imaging of POS-UCNPs/ICG metabolism in *vivo* (Control group was injected with PBS). (D) Histological images of H&E staining of major organs in BALBC/c mice 60 days after injection.

To explore the feasibility of POS-UCNPs/ICG as an antimicrobial and anti-inflammatory agent for clinical application, biosafety assay was conducted by CCK-8 activity of L-929 cells, fluorescence imaging of metabolism in *vivo* and the long-term toxicity in *vivo* upon the challenging of POS-UCNPs/ICG composites. As shown in Figure 4A, cell activity gradually decreased with the increase of POS-UCNPs/ICG concentration. Compared with the control group (0 µg·mL^-1^), the cell viability of POS-UCNPs/ICG was below 80% with the concentration of POS-UCNPs/ICG increased up to 200 µg·mL^-1^. Obviously, POS-UCNCPs/ICG cell survival rate was above 80% (with NIR light) and 90% (without NIR light) at 100 μg·mL^-1^, respectively, showing satisfied biosafety of 100 μg·mL^-1^ POS-UCNCPs/ICG. In addition, FITC/DAPI staining in human gingival fibroblasts (HGFs) were further used to estimate the cytotoxicity of POS-UCNPs/ICG (Figure S18). This result was consistent with cell survival, with only a few round cells appearing up to 200 µg·mL^-1^, which is considered necrotic cells.

Further, fluorescence imaging in *vivo* was used to assess POS-UCNPs/ICG metabolism. As shown in Figure 4B and S19A, obvious fluorescence could be seen in mice at 1 h after intravenous injection. However, fluorescence decreased significantly at 24 h after injection, indicating that POS-UCNPs/ICG could be rapidly metabolized from mice. To demonstrate the detailed metabolic processes of the mice, the organs were collected and imaged at different time points. Figure 4C and S19B showed that POS-UCNPs/ICG fluorescence was mainly concentrated in liver and kidney at 1 h post injection. After 24 hours of metabolism, there was no fluorescent signal in other organs except for some residue on the liver. We collected feces from mice at 24 h post injection. This fluorescence was much stronger than that of the control group (Figure. S19C-D), demonstrating that mice could metabolize POS-UCNPs/ICG through feces. Finally, the long-term toxicity of POS-IUCNPs/ICG was assessed in mice. As shown in Figure 4D, major organs were collected 60 days after injection and histological analysis of hematoxylin and eosin (H&E) in the organs showed no abnormalities or inflammation. These results indicated that POS-UCNPs/ICG at 100 µg·mL^-1^ had good biosafety and great potential for application in *vivo*.

### Antibacterial activity of POS-UCNPs/ICG *in vitro*

**Figure 5:**
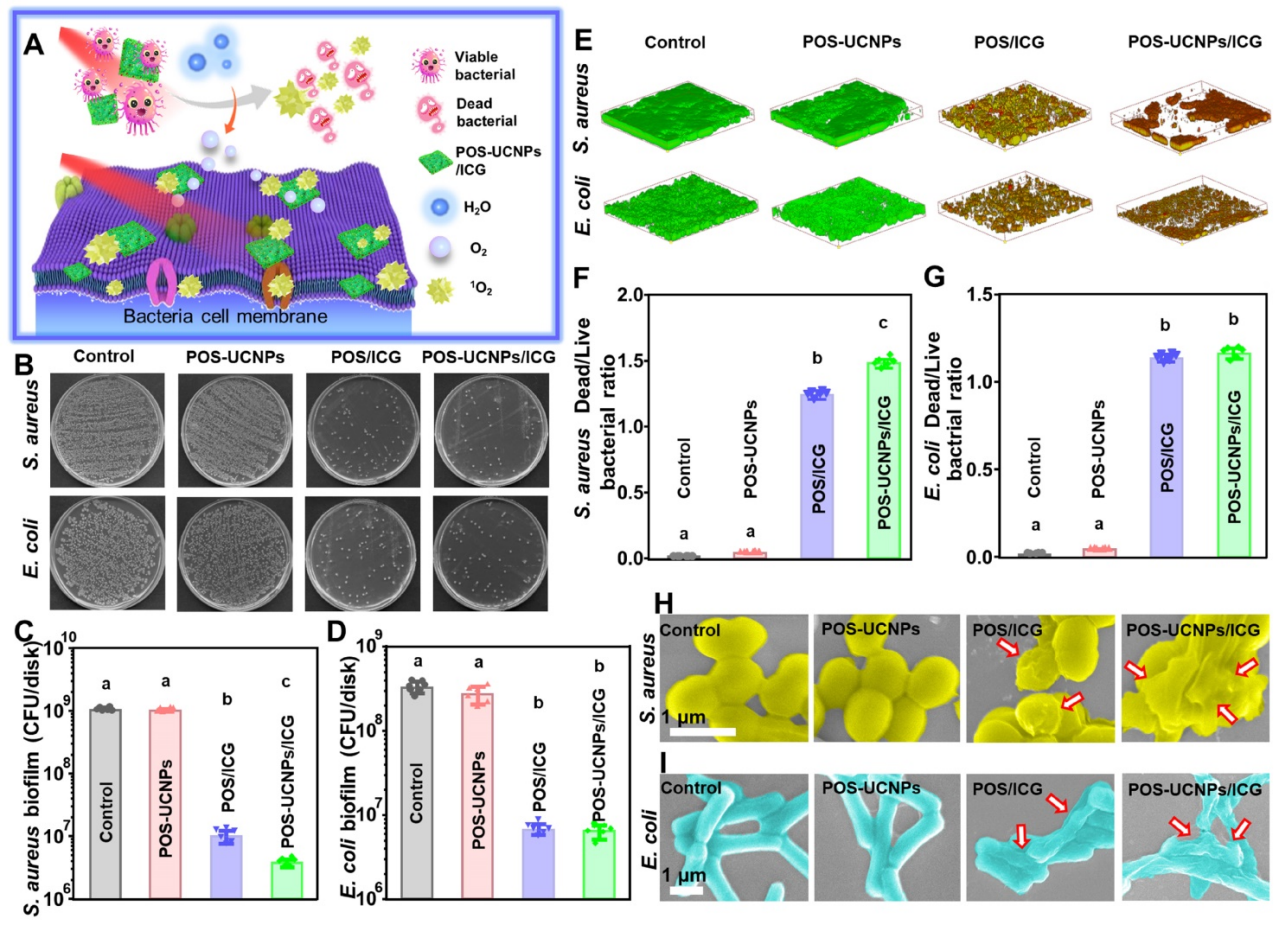
The anti-biofilm ability of POS-UCNPs/ICG. (A) Schematic illustration of enhancing aPDT by POS-UCNPs/ICG. (B) The colony formation images of *S. aureus* and *E. coli* after treatments with different nanomaterials. CFU counts of (C) *S. aureus* and (D) *E. coli-*biofilms with different treatments. (E) 3D dead/live images of biofilms of *S. aureus* and *E. coli* at 4 days (dead bacteria stained red; live bacteria stained green). Dead/live bacteria ratio for (F) *S. aureus* and (G) *E. coli*. The SEM images of (H) *S. aureus* and (I) *E. coli* with different treatments (The red arrows indicated the folding and deformation of the bacterial membrane). Dissimilar letters represented statistical differences between each other (n = 6, p < 0.05).

In healthy tissues, the O_2_ tension was reported at about 144 mm Hg, while infection sites showed much lower oxygen levels of about 28 mm Hg 49. Such hypoxic microenvironment in infection sites would significantly impede the function of aPDT to combat bacteria. Fortunately, POS-UCNPs/ICG could convert H_2_O into O_2_, which was beneficial for improving the therapeutic effect of aPDT (Figure 5A). Based on the above assumption, single strain biofilm was used to test the antimicrobial properties of POS-UCNPs/ICG.

In the antimicrobial evaluation of biofilms, *S. aureus* and* E. coli* were selected as Gram-positive and Gram-negative representative strains to form single-species biofilms to investigate the antimicrobial properties of POS-UCNPs/ICG. The bacterial survival rate of *S. aureus* and *E. coli* decreased to zero, when the POS-UCNPs/ICG concentration reached 50 μg·mL^-1^, proving that the minimum inhibitory concentration (MIC) of the nanoplatform was 50 μg·mL^-1^ (Figure S20). However, the therapeutic effect of *S. aureus* is better than that of *E. coli* at 6.25 μg·mL^-1^, 12.5 μg·mL^-1^ and 25 μg·mL^-1^. Figure 5B plotted the colony formation units (CFU) counts of *S. aureus* and *E. coli* upon different challenges in presence of light irradiation. Compared with the control group, the biofilm CFU of *S. aureus* and *E. coli* in POS-UCNPs group was not significantly reduced (p > 0.05), indicating the limited anti-biofilm capacity of CO. Interestingly, Cai et al. considered CO gas exhibited antibacterial activity 50. The POS-UCNPs group with light irradiation had no therapeutic effect in the present study, because the produced CO concentration was not high enough to destroy bacterial biofilms 51. Herein, the biofilm only produced approximately 89 mm Hg CO_2_ pressure, thus resulting in insufficient CO to fight bacteria 49. Remarkably, biofilm CFU in POS/ICG group and POS-UCNPs/ICG group were significantly decreased in contrast to the control group (p < 0.05). As plotted in Figure 5C, *S. aureus* in the POS/ICG and POS-UCNPs/ICG group underwent a respective reduction of 2 and 3 orders of magnitude, when comparing with control group. Interestingly, ICG not only has photothermal effect but also aPDT function. Therefore, the photothermal effect of POS-UCNPs/ICG was studied (Figure S21). The results showed that the temperature changes only about 5 ℃ within 5 min, because part of the NIR excitation energy was absorbed by POS-UCNPs NSs for gas production. Therefore, we believed that the photothermal effect of POS-UCNPs/ICG was not so obvious in this work. These results indicated that antibacterial activity was mainly due to ICG-mediated aPDT function. The different killing efficiency between POS/ICG and POS-UCNPs/ICG demonstrated that the introduction of UCNPs would converse 808 nm NIR to the green light, thus triggering POS NSs to decompose H_2_O to generate O_2_ for aPDT enhancement (Figure 5C). On the contrary, *E. coli* in the POS/ICG group and POS-UCNPs/ICG groups showed similar reduction of less 2 orders of magnitude compared with control group (Figure 5D), since Gram-negative bacteria possessed outer cell membrane containing LPS that blocked connection PSs with cell membrane protected the Gram-negative bacteria from ROS attacking 52, 53. The similar results were also found in the dead/live staining assay (Figure 5E). The dead bacteria with red staining were significantly increased in the POS/ICG and POS-UCNPs/ICG groups. Notably, POS-UCNPs/ICG group had the highest ratio of dead/live bacteria of *S. aureus*, indicating the excellent anti-biofilm capacity of POS-UCNPs/ICG nanoplatform upon NIR irradiation (Figure 5F-G).

Furthermore, the alteration of bacterial morphology upon POS-UCNPs/ICG treatment was also evaluated by SEM observation. As shown in Figure 5H-I, *S. aureus* exhibited a smooth and intact cell membrane in the control group. Bacteria with POS-UCNPs treatment under NIR irradiation treatment had no obvious change. While, POS/ICG with NIR irradiation treatment led to the slight shrinkage of *S. aureus* membrane. Interestingly, POS-UCNPs/ICG with NIR irradiation treatment led to the most severe damage of *S. aureus* cell membrane. The morphological change of *E. coli* upon different treatments showed an identical trend. The aforementioned results were also consistence with the metabolic activity of bacterial biofilm as shown in Figure S26. In addition, the unilluminated groups had the similar results as those of the control groups (Figure S22-26), proving that light and nanomaterials alone had rarely therapeutic effect on killing bacterial biofilm.

### Anti-inflammation of POS-UCNPs/ICG *in vitro*

**Figure 6:**
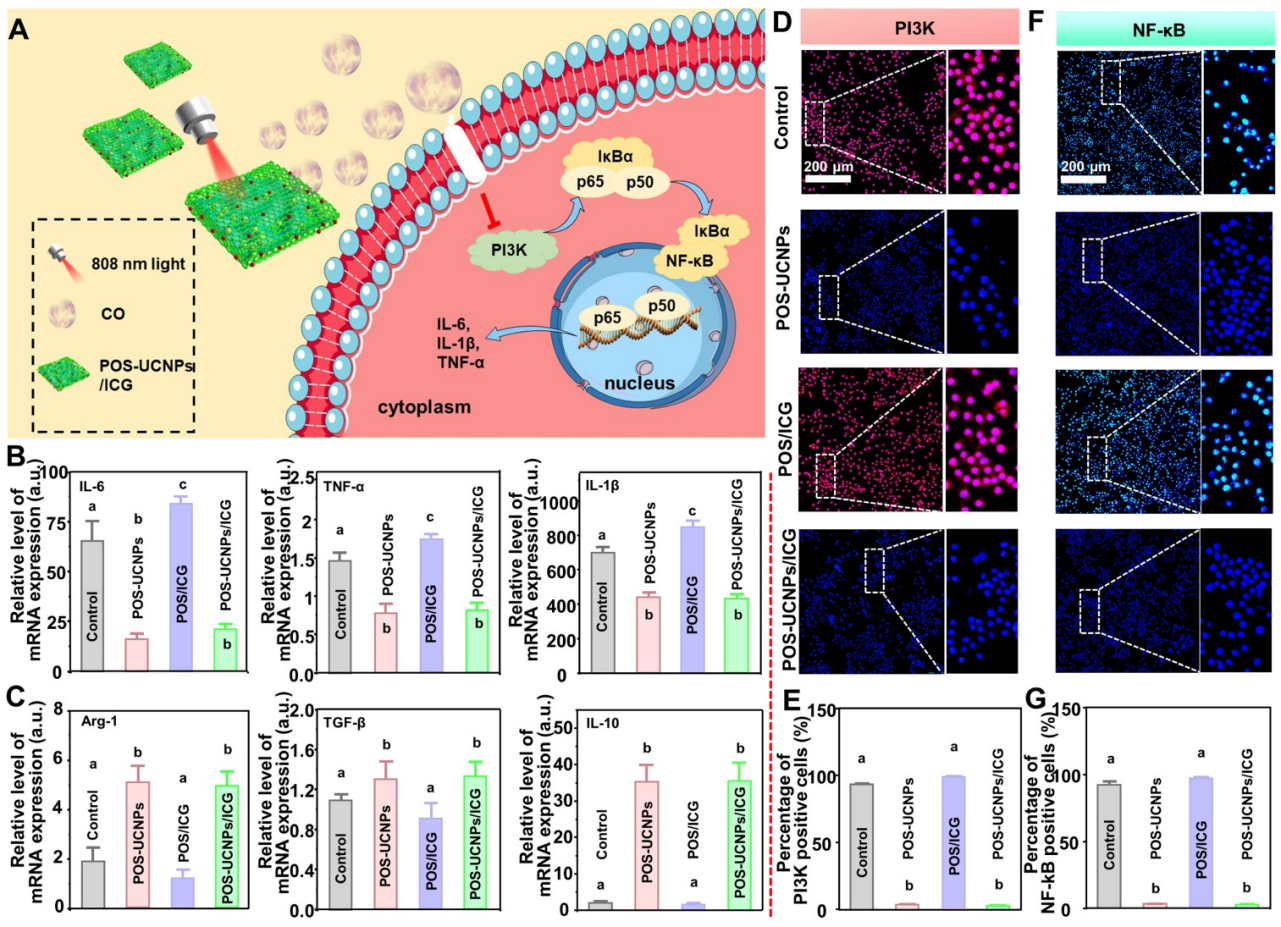
Anti-inflammation of POS-UCNPs/ICG by inhibiting PI3K/NF-κB signaling pathways. (A) Schematic diagram of anti-inflammation mechanism of POS-UCNPs/ICG by producing CO. (B) Gene expression of M1 biomarkers (TNF-α, IL-1β and IL-6) and (C) Gene expression of M2 biomarkers (Arg-1, TGF-β, and IL-10) after stimulation (The values of the no-stimulus group were normalized to 1). (D) Immunofluorescent images of PI3K/p110 in macrophages. (E) The quantification of p110 macrophages in Figure D. (F) Immunofluorescent images of macrophages NF-κB/p65 translocation. (G) The rate of positive cells in Figure F. Dissimilar letters represented statistical differences between each other (n = 6, p < 0.05).

In bacteria-induced infectious diseases, bacteria manipulate the inflammatory responses, inducing M0 macrophages towards M1 polarization. However, prolonged M1 polarization would secrete destructive pro-inflammatory cytokines, ultimately resulting in tissue injury. Even the bacteria were eliminated, the M1 polarized macrophages could not immediately reverse. Therefore, inflammation regulation was another key point for the clinical application of nanomedicine for bacteria-induced infectious diseases. As mentioned above, CO at a lower concentration had nearly no antibacterial effects against infections. However, CO at a such low level could serve as an endogenous signal molecule for the inhibition local inflammation 54. As reported, the low concentration of CO was involved into the nuclear factor kappa B (NF-κB) and PI3K pathways, regulating macrophage polarization 55,56. Therefore, POS-UCNPs/ICG could convert CO_2_ to CO by photocatalysis, theoretically modulating macrophage polarization via the PI3K-mediated NF-κB pathway for anti-inflammation (Figure 6A).

The* E. coli's* LPS, a component of the cell wall of Gram-negative bacteria, was used to stimulate macrophages to mimic an inflammatory microenvironment *in vitro* 54. To investigate whether POS-UCNPs/ICG could reverse macrophages polarization, we tested key pro-inflammatory factors (IL-1β, IL-6 and TNF-α) and anti-inflammatory factors (IL-10, Arg-1 and TGF-β) after various treatments. As shown in Figure 6B-C, POS-UCNPs showed strong reverse regulation ability to LPS-induced pro-inflammatory factor overexpression under the irradiation of 808 nm light, providing that CO could inhibit bacterial LPS-induced transformation of macrophages to M1 type. Subsequently, pro-inflammatory factors were further upregulated in the POS/ICG group compared with the control group under 808 nm light irradiation, attributing to the ROS production. Such elevation of pro-inflammatory factor expression would further reduce due to the fact that the introduction of UCNPs in POS-UCNPs/ICG would facilitate the generation of CO in local microenvironment. Macrophages displayed different polarization states because of changes in the microenvironment. M1 type macrophages, which is closely related to inflammation, aggregates to the infection site and plays the role of endogenous immune system 57. Bacteria induced-M1 macrophages increase pro-inflammatory factors, damaging the surrounding normal tissue. While M2 type macrophages, which are closely related to anti-inflammation, participate in tissue regeneration and wound healing through oxidative metabolism 58. Importantly, the CO signal molecules generated in POS-UCNPs and POS-UCNPs/ICG could also up-regulate the anti-inflammatory factors of M2-subtype macrophages, indicating that CO could not only inhibit local inflammation, but also promote the tissue regeneration.

NF-κB signaling pathway was prominent in inflammatory regulation. NF-κB signaling pathway could be activated by LPS, followed pro-inflammatory factors up-regulation. Translocation of NF-κB/p65 subunit was a key process in the activation of the NF-κB signaling pathway, so it was important to inhibit the nuclear translocation of NF-κB after activation 59, 60. PI3K was one of the major upstream factors of NF-κB activation. The inhibition of PI3K could remarkably reduce NF-κB/p65 subunit phosphorylation, which further suppressed the DNA-binding activity of NF-κB 61-65. To verify that CO could achieve anti-inflammatory purpose through PI3K/NF-κB signaling pathway, we performed fluorescence staining for upstream factor PI3K and downstream factor NF-κB. Firstly, the upstream factor PI3K/p110 was stained with red fluorescence. As shown in Figure 6D, there was almost no p110 positive cells in POS-UCNPs and POS-UCNPs/ICG groups with light irradiation in comparison to POS/ICG and control groups with positive cells in almost full sights. The number of p110 positive cells in the POS/ICG and control groups had a significantly higher intensity under NIR light than that in POS-UCNPs and POS-UCNPs/ICG groups (Figure 6E). Afterwards, fluorescence staining of downstream factor NF-κB/p65 was also performed. As shown in Figure 6F, the translocation of NF-κB/p65 took place in POS/ICG and control group. However, the number of p65 positive cells in POS-UCNPs and POS-UCNPs/ICG groups was significantly decreased (Figure 6G). Taken Together, CO molecules could inhibit the p65 subunit phosphorylation of NF-κB by suppressing the activity of PI3K and then restraining the DNA-binding activity of NF-κB, finally achieving the anti-inflammatory effect. Figure S27-29 showed the results of the groups without light irradiation, proving light and nanomaterials alone had no anti-inflammatory effect.

### Antibacterial and anti-inflammation of POS-UCNPs/ICG *in vivo*

**Figure 7:**
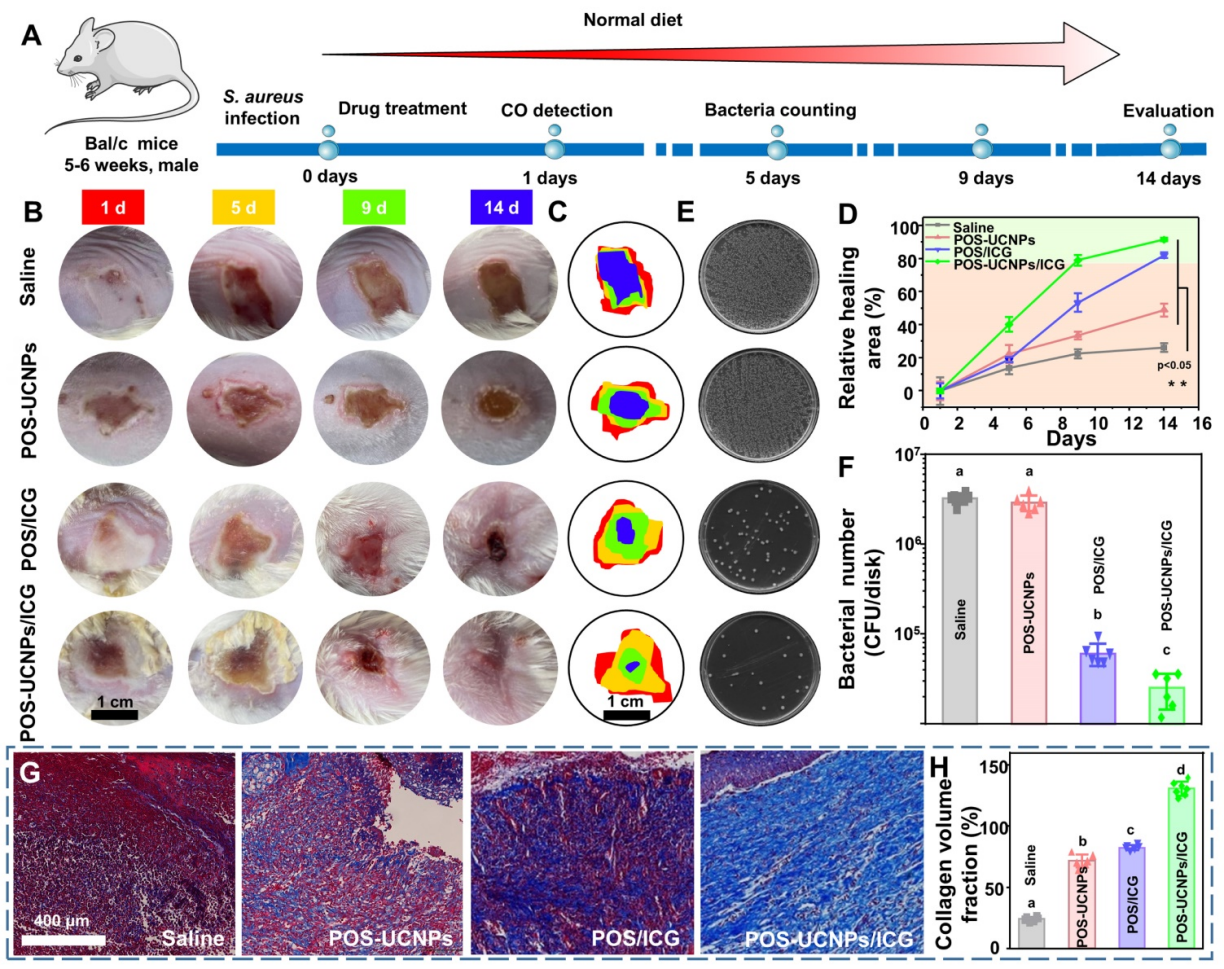
The *in vivo* therapeutic efficacy of POS-UCNPs/ICG on treating subcutaneous abscess of mice. (A) Schematic diagram of *in vivo* therapeutic protocol. (B) Digital photos of subcutaneous abscess at different time points after various treatments. The illustration with different colors represented different days. (C) Variation in the size of abscess areas of Saline, POS-UCNPs, POS/ICG and POS-UCNPs/ICG groups. (D) Relative area of healing after various treatments. (E) Photographs of bacterial colonies from different abscesses after various treatments. (F) The number of bacteria after various treatments. (G) Masson staining images of various treated abscess tissues. (H) The relative collagen volume in Masson staining sections. Dissimilar letters represented statistical differences between each other (n = 6, p < 0.05).

Inspired by the efficient properties of anti-biofilm and anti-inflammation* in vitro*, the capacity of POS-UCNPs/ICG in treating bacterial infections were further assessed in a subcutaneous abscess model. The subcutaneous abscess model was constructed by local injection of *S. aureus* 19. The detailed experimental programs, from the establishment of the infection model to drug administration and related evaluations, were presented in Figure 7A. Briefly, *S. aureus* (10^8^ CFU·mL^-1^,100 μL) were injected subcutaneously into the shaved backs of the BALB/c mice. After injecting bacteria for 5 min, the mice in different groups were subcutaneously injected with saline, POS-UCNPs, POS/ICG, and POS-UCNPs/ICG (100 μg·mL^-1^, 0.01 mL per gram of body weight) into the backs following by 808 nm light irradiation (1 W·cm^-2^, 5 min). The healing conditions of the abscess site were systematically recorded via macroscopic images analysis. As depicted in Figure 7B, the abscesses in the saline group demonstrated the most inferior healing conditions without any nanomedicine intervention. POS-UCNPs with sole CO therapy and POS/ICG with sole aPDT function exhibited a more favorable healing process. Interestingly, when CO therapy combined with aPDT, POS-UCNPs/ICG group showed superior therapeutic effects, displaying scab formation at 5 days and well wound healing at 14 days. The synergistic effects of anti-biofilm capacity and anti-inflammation by POS-UCNPs/ICG would potentially rehabilitate the local abscess by killing bacterial pathogens and relieving the dramatic immune response. The abscess size during the healing process was recorded and analyzed in Figure 7C-D. The percentages of healing area in the saline and POS-UCNPs groups were 26.15±3.26% and 48.91±5.65% respectively. Whereas, the healing area of POS/ICG group were remarkably reach more than 80%. In particular, POS-UCNPs/ICG groups had a highest percentage of healing area up to 91.55±1.26%. Notably, the POS/ICG group exhibited a greater progress in healing process than POS-UCNPs group, due to the efficient sterilization of POS/ICG to avoid excessive immune for tissue damage. In the initial phase of bacterial infection, the immune response is induced to facilitate the removal of bacteria 66. Once the intruders are not cleared, the immune response are over-reactive, leading to damage the tissue 67. Thereby, bacteria elimination is more critical for the healing process 68, 69.

Accordingly, as shown in Figure 7E-F, CFU counts plotted that the POS/ICG and POS-UCNPs/ICG groups could excellently eradicate biofilms. The lowest CFU counts in POS-UCNPs/ICG group was attribute to the enhanced aPDT of O_2_ production. Finally, the status of collagen fibers (blue staining) during the healing process at the abscess site was monitored by Masson trichrome staining (Figure 7G and S30). The saline group exhibited a severe collage collapse and destruction during the whole pathogenic process without any treatment. On the contrary, the abscess with different treatment had an accelerated healing in different degree. Remarkably, the abundant and well-organized collagen fibers were observed in the POS-UCNPs/ICG group. The collagen fiber volume in the POS-UCNPs/ICG group was the largest among all the experimental groups (Figure 7H.).

**Figure 8:**
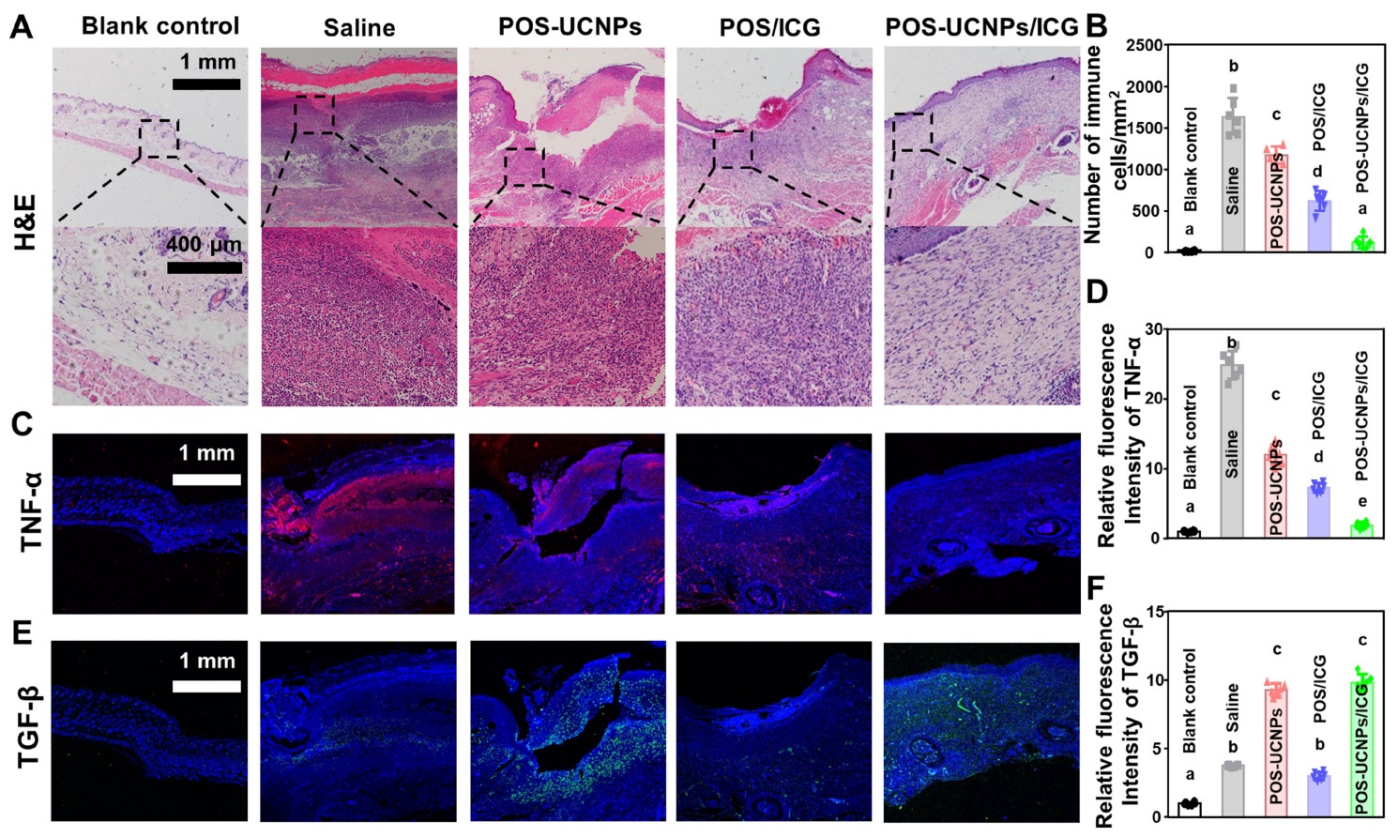
Evaluation of immunomodulatory effect of POS-UCNPs/ICG *in vivo*. (A) H&E staining of various treated abscess tissues. (B) Number of immune cells (monocytes, macrophages, neutrophils and lymphocytes) in HE samples. (C) Immunofluorescence images of the infection sites. TNF-α positive cells were stained with red fluorescence. (D) The relative immunofluorescence intensity of TNF-α positive cell. (E) Immunofluorescence images of TGF-β expressed in abscess. TGF-β positive cells were visualized by green fluorescence. (F) The relative fluorescence intensity of TGF-β positive cells. Dissimilar letters represented statistical differences between each other (n = 6; p < 0.05).

The local CO level in POS-UCNPs/ICG and POS-UCNPs groups were greatly increased compared to saline group due to the CO releasing upon 808 nm NIR irradiation in the presence of such nanomaterials (Figure S31), which probably played a significant role in tissue immunoregulation *in vivo* 58. Figure S31 showed that CO content in POS-UCNPs and POS-UCNPs/ICG groups increased from 21.55 pmol·mg^-1^ to 62.16 pmol·mg^-1^, which was close to the 70 pmol·mg^-1^ CO that has been reported to achieve an anti-inflammatory effect 39. Then the histological alteration at abscess site was verified by H&E staining (Figure 8A). The tissue around in the saline group displayed severe infiltration of inflammatory cells, such as neutrophils and macrophages. While, inflammatory cells slightly decreased in POS-UCNPs and POS/ICG groups due to the CO production of POS-UCNPs and the biofilm eradication of POS/ICG. Similarly, POS-UCNPs/ICG group showed rare infiltration of inflammatory cells (7.31 ± 3.66% vs. saline group, Figure 8B). The aPDT could effectively kill the bacteria to alleviate the local inflammation. However, the excessive ROS also could further induce the immune response, delaying the healing process 70, 71. Fortunately, POS-UCNPs/ICG upon NIR irradiation could simultaneously exert the potent killing efficiency against bacterial pathogens by aPDT function, subsequently modulate the local inflammation by the elevated level of CO gas. Ultimately, to further observe the immunoregulatory effects on promoting abscess recovery, the expression of cytokine at the abscess site were analyzed by immunofluorescence staining and real-time PCR assay.

As shown in Figure 8C, TNF-α positive cells with red fluorescence were negatively detected in blank control. While, substantial TNF-α positive cells was observed in the saline group with *S. aureus* stimulated abscess. Similar with blank control, TNF-α positive cells was also scarcely observed in POS-UCNPs/ICG group. The relative fluorescence intensity of TNF-α in the POS-UCNPs/ICG group reduced back to the similar level with blank control group (1.89 times fluorescence intensity vs. blank control, Figure 8D). On the other hand, the green fluorescence-stained TGF-β, as a representative anti-inflammatory factor, was displayed in the POS-UCNPs and POS-UCNPs/ICG group (Figure 8E). The results of quantitative analysis showed the high expression level of TGF-β in POS-UCNPS and POS-UCNPs/ICG groups (Figure 8F), due to the immunoregulatory capacity of CO gas. The similar trend was also detected by real-time PCR assay. The expression of IL-1β and TNF-α in POS-UCNPs/ICG group were significantly down-regulated compared with those in the other groups. Meanwhile, TGF-β and IL-10 were obviously up-regulated (Figure S32). Therefore, integrated with its potent anti-biofilm capacity, POS-UCNPs/ICG could restrain bacteria invasion, and regulate the immune respond for potentially accelerating the recovery of bacteria induced abscess *in vivo*.

## Conclusion

In this study, a NIR light responsive nanoplatform (POS-UCNPs/ICG) was designed for CO/enhanced aPDT synergistic treatment of bacteria-induced infection diseases. POS-UCNPs/ICG was built using the POS-UCNPs NSs, which was triggered with NIR light for CO and O_2_ production via photo-catalyze and provided a carrier for the ICG. It was noteworthy that POS-UCNPs/ICG could enhance the aPDT against bio-film with the decomposition H_2_O for O_2_ production. More importantly, POS-UCNPs/ICG could product CO gas to facilitate the immunoregulation, while efficient elimination biofilms, thereby outstanding promoting tissue recovery. Considering the synergistic anti-biofilm capacity and immunoregulation of POS-UCNPs/ICG, the NIR light responsive nanoplatform represented a promising strategy for bacteria-induced infection diseases.

## Supplementary Material

Supplementary methods, figures and tables.
