# Supplementary material for: Antibacterial PDT nanoplatform capable of releasing therapeutic gas for synergistic and enhanced treatment against deep infections
Source: Theranostics. 2022 Feb 28;12(6):2580–97. doi: 10.7150/thno.70277 (PMC8965476; doi:10.7150/thno.70277)
Supplement: Supplementary file 1 — Supplementary methods, figures and tables. [file thnov12p2580s1.pdf]

Supporting Information

Antibacterial PDT Nanoplatform Capable of Releasing Therapeutic Gas for Synergistic and Enhanced Treatment against Deep Infections

Bingshuai Zhou[1#], Xiaolin Sun[2#], Biao Dong[1*], Siyao Yu[1], Liang Cheng[2], Songtao Hu[1], Wei Liu[1], Lin Xu[1], Xue Bai[1], Lin Wang[2*], Hongwei Song[1*]

[1] State Key Laboratory on Integrated Optoelectronics, College of Electronic Science and Engineering, Jilin University, Changchun, 130012, China;

[2] Department of Oral Implantology, Jilin Provincial Key Laboratory of Sciences and Technology for Stomatology Nanoengineering, Hospital of Stomatology, Jilin University, Changchun, 130021, China;

***Correspondence:**

Prof. Hongwei Song, Email: songhw@jlu.edu.cn;

Prof. Lin Wang, Email: wanglin1982@jlu.edu.cn;

Prof. Biao Dong, Email: dongb@jlu.edu.cn.

#These authors contribute equally to this work.

1. Additional Experimental Methods

1.1 Preparation of CTAB modified UCNPs. CTAB modified UCNPs were prepared via a traditional approach. Briefly, 5 mL UCNPs cyclohexane solution was added to an aqueous solution containing 0.1 g CTAB and 20 mL DI water. Then the mixtures were stirred vigorously with 70 °C until the cyclohexane solvent was fully evaporated, forming UCNPs-CTAB water solution.

1.2 In vitro CO detection. The 2 mL saturated sodium bicarbonate solution containing POS-UCNPs/ICG 200 μg, CO probe (5 μM) and $PdCl_2$ (5 μM) was put placed in a cuvette. The fluorescent spectra was recorded at a time every 5 min under 808 nm (1 $W\cdot cm^{-2}$) light irradiation. The excitation wavelength of CO probe was 488 nm.

1.3 In vitro O_2 detection. 2 mL aqueous solution containing 200 μg POS-UCNPs/ICG and 620 ng O_2 probes was put into a cuvette and injected nitrogen for 30 min in order to remove the O_2 in solution. The fluorescent spectra were recorded after a few min of 808 nm laser irradiation. The excitation wavelength of O_2 probe was set at 455 nm.

1.4 In vitro ROS detection. 100 $\mu g\cdot mL^{-1}$ POS-UCNPs/ICG and 50 $\mu g\cdot mL^{-1}$ 1,3-Diphenylisobenzofuran (DPBF) in 2 mL of aqueous solution was put into a cuvette. The absorption spectra were recorded after per min of 808 nm light irradiation.

1.5 Cell culture and cytotoxicity assay. L-929 cells were cultured in an incubator at 37 °C with 5% CO_2 and incubated in DMEM medium containing 1% penicillin-streptomycin (100 $U\cdot mL^{-1}$ penicillin and 100 $g\cdot mL^{-1}$ streptomycin) and 10% fetal bovine serum (FBS, Clark). Human gingival fibroblasts (HGFs) were co-incubated with different concentrations of POS-UCNPs/ICG for 24 h to evaluate cytotoxicity. Next, the cells were washed with PBS and stained with fluorescein isothiocyanate (FITC)/4',6-diamidino-2- phenylindole (DAPI) for 15 min in a dark environment. Finally, the cells were imaged in confocal laser scanning microscope (CLSM; FV1000, Olympus).

1.6 CO detection. L-929 cells were co-culture with PBS (control), POS-UCNPs, POS/ICG and POS-UCNPs/ICG (100 $\mu g\cdot mL^{-1}$) for 4 h. Then the cells were cultured in a fresh medium containing CO probe (5 μM) and $PdCl_2$ (5 μM). After the cells were irradiated by 808 nm laser (1 $W\cdot cm^{-2}$) for 5 min, the cells were incubated at 37 °C for 30 min. Next, the cells were washed with PBS and imaged by CLSM.

1.7 ROS detection. L-929 cells were co-culture with PBS (control), POS-UCNPs, POS/ICG and POS-UCNPs/ICG (100 μg·mL^{-1}) for 4 h. Then the cells were cultured in a fresh medium that contained 10 μM of 2, 7-Dichlorodihydrofluorescein diacetate (DCFH-DA). After irradiated by 808 nm laser (1 W·cm^{-2}, 5 min), the cells incubated at 37 °C for 20 min. Next, the cells were washed with PBS three times and imaged by CLSM.

1.8 Preparation of different mass ratio POS-UCNPs. The synthesis method was same as POS-UCNOPs in the article, except for changing the mass ratio of $SnCl_4·5H_2O$

1.9 Preparation of different mass ratio POS-UCNPs/ICG. The synthesis of POS-UCNPs followed with the section 2.3 in the text. Subsequently, different mass ratios of ICG and POS-UCNPs (1:5, 1:10 and 1:15) were added into the reaction system and stirred for 12 h at room temperature. The nanoparticles were washed three times with DI water and afterward centrifugated at 9000 rpm for 5 min.

1.10 Detection of loading rate and encapsulation rate. The drug concentrations of the initial solution and the supernatant were measured according to the ICG UV-vis absorption spectrum at 780 nm. The load rate and *encapsulation* rate of ICG are calculated by the following equation:

$$\text{Loading rate} = (W_i - W_{\text{Non-encapsulated}})/W_{\text{total}} \times 100\% \qquad (1)$$

$$\text{Encapsulation rate} = (W_i - W_{\text{Non-encapsulated}})/W_i \times 100\% \qquad (2)$$

Where W_i represents the initial total mass of added drug; $W_{\text{Non-encapsulated}}$ is the unencapsulated drug mass in supernatant after centrifugation; W_{total} represents the total mass of added POS-UCNPs nanosheets (NSs) and encapsulated ICG drug.

1.11 The quantitative detection of CO. Reduced hemoglobin was used to test CO produced by POS-UCNPs/ICG. In short, excess (0.1%) sodium dithionite solution was added to a 20 μg·mL^{-1} reduced hemoglobin solution with 500 μL deoxygenated PBS. After that, the mixture was purged with nitrogen for 10 min. Afterward, POS-UCNPs/ICG suspension with 500 μL deoxygenated PBS was added into aforementioned mixtures. With NIR irradiation (1 W·cm^{-2}) for 25 min, the UV-vis

absorption spectra were recorded by an ultraviolet spectrophotometer. The concentration of produced CO (C_{CO}) was calculated by following equation:

$$C_{CO}=\frac{528\times(I_{410})-304\times(I_{430})}{216.5\times(I_{410})+442.4\times(I_{430})}\times C_{Hb};$$

where, C_{CO} and C_{Hb} represented the produced CO concentration and the used Hb concentration, respectively. I_{410} and I_{430} were the absorbance at the wavelength of 410 and 430 nm, respectively."

1.12 Minimum inhibitory concentration (MIC) of POS-UCNPs/ICG. *S. aureus* and *E. coli* were cultured in TSB medium at 37 °C for 4-6 h at a speed of 200 rpm. When the absorption value of bacterial solution at 600 nm was 0.1, the corresponding bacterial concentration was 1×10^{8} CFU·mL^{-1} (diluted with broth). A bacterial suspension of 20 μL1×10^{9} CFU·mL^{-1} was added to 160 μL PBS. Then PBS and POS-UCNPs/ICG were added to 96-well plates and cultured at 37 °C for 18 h on a shaker bed. Then the value of the 96-well plate at OD_{600nm} were detected by the microplate reader.

1.13 The metabolic activity analysis of biofilms. *S. aureus* and *E. coli* biofilms were respectively co-incubated with MTT for 1 h, and then the biofilms were treated with DMSO in order to dissolve formazan crystals. Next, the tinfoil coated 24-well plate was shaken on a shaker for 20 min. Then the solution was moved to the 96-well plate and the luminosity value at 540 nm was measured by a microplate reader.

1.14 Detection of CO in abscess tissues. Mice bearing *S. aureus*-induced abscesses were subcutaneously injected with 200 μL of saline, POS-UCNPs, POS/ICG, and POS-UCNPs/ICG (100 μg·mL^{-1}). Then 808 nm light irradiation (1 W·cm^{-2}) was given at the abscess tissue site for 5 min. Next, mice were euthanasia, and the abscess tissue was rapidly acquired for CO detection via endogenous carbon monoxide assay kit (A101−2−1).

1.15 ***Cell cytokines detection in vivo.*** After euthanasia, all infected tissue were removed and placed on RNase free tubes and stored in liquid nitrogen. Next, the tissues were ground into powder in the presence of liquid nitrogen and the RNA were isolated using Trizol. The following procedure was the same as the in vitro qPCR procedure.

2. Supplementary table

Table s1. Primer sequences used in this study

Gene	Forward Sequence (5'to3')	Reverse Sequence (5'to3')
β-actin	CATCCGTAAAGACCTCTAGCCAAC	ATGGAGCCACCGATCCACA
IL-1β	TCCAGGATGAGGACATGAGCAC	GAACGTCACACACCAGCAGGTTA
IL-6	CCACTTCACAAGTCGGAGGCTTA	CCAGTTTGGTAGCATCCATCATTTC
TNF-α	ACTCCAGGCGGTGCCTATGT	GTGAGGGTCTGGGCCATAGAA
TGF-β	CTTCAGCCTCCACAGAGAAGAACT	TGTGTCCAGGCTCCAAATATAG
Arg-1	TGTGTCCAGGCTCCAAATATAG	AGCAGGTAGCTGAAGGTCTC
IL-10	ATGCTGCCTGCTCTTACTGACTG	CCCAAGTAACCCTTA AAGTCCTGC

Table S2. Content of elements of the surface of POS-UCNPs NSs (in at% from XPS analysis)

	XPS (at%)								
Elements	Sn	S	Na	Y	F	Yb	Er	Nd	O
	8.43	31.14	0.65	1.78	9.55	0.84	0.6	2.54	44.47

3. Supplementary figures

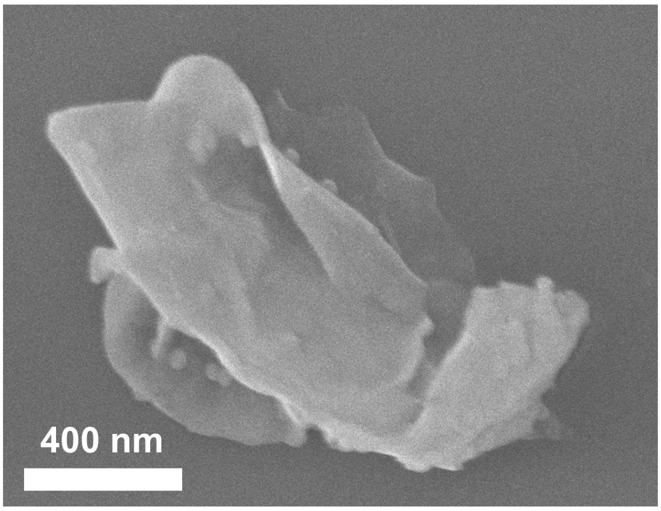

Figure S1. SEM image of POS NSs.

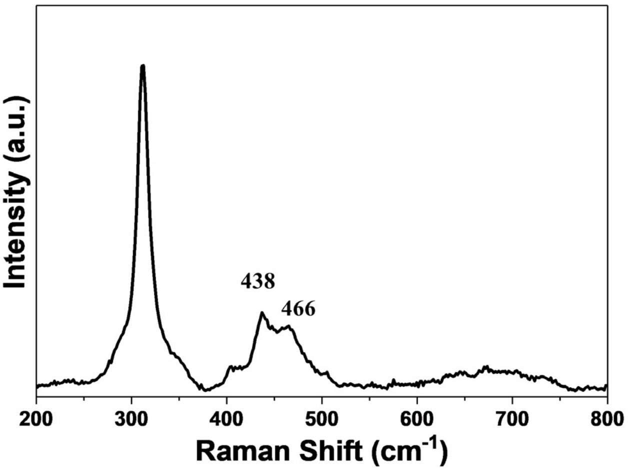

Figure S2. Raman spectrum for POS NSs excited by 532 nm laser. The peaks at 438 cm^{-1} and 466 cm^{-1} demonstrated the presence of SnO_2 phase in POS NSs.

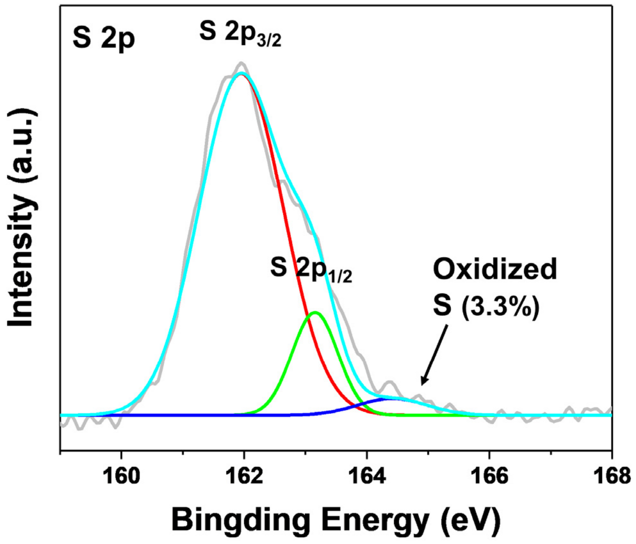

Figure S3. S 2p XPS spectrum for POS NSs. The oxidized S was detected to be 3.3%.

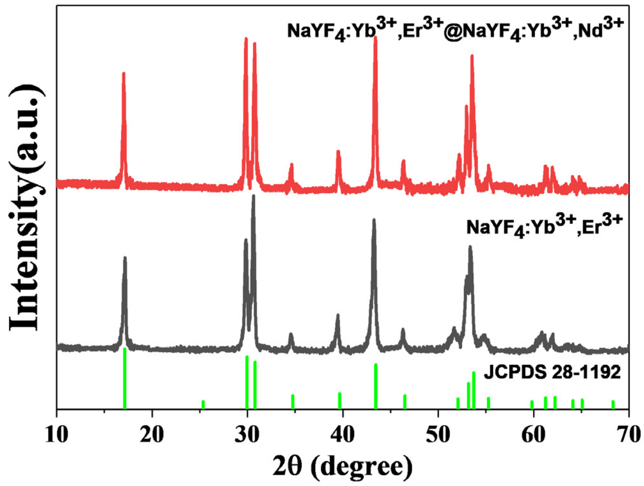

Figure S4. XRD pattern of $NaYF_4:Yb^{3+},Er^{3+}$ and $NaYF_4:Yb^{3+},Er^{3+}@NaYF_4:Yb^{3+},Nd^{3+}$.

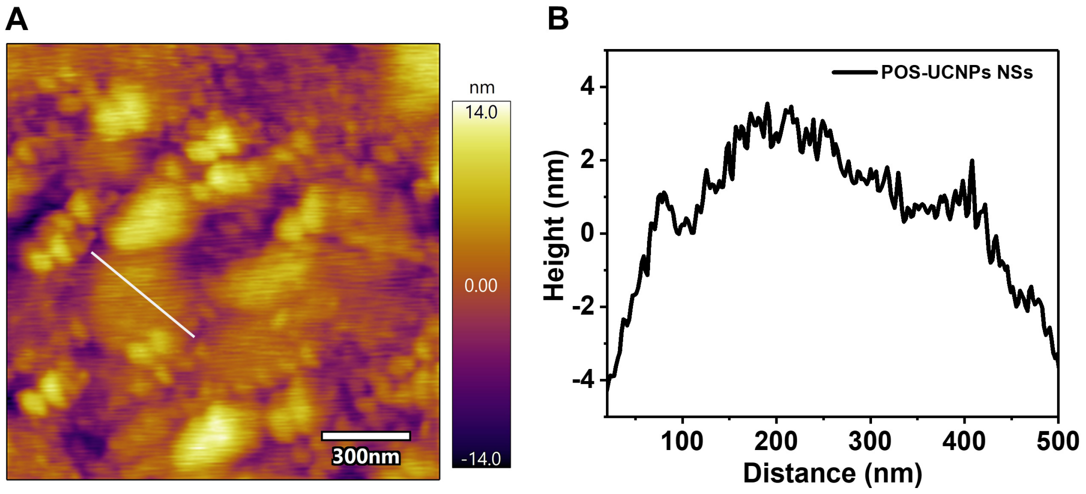

Figure S5. (A) Atomic force microscopy (AFM) image of POS-UCNPs NSs. (B) Corresponding height profile of POS-UCNPs NSs

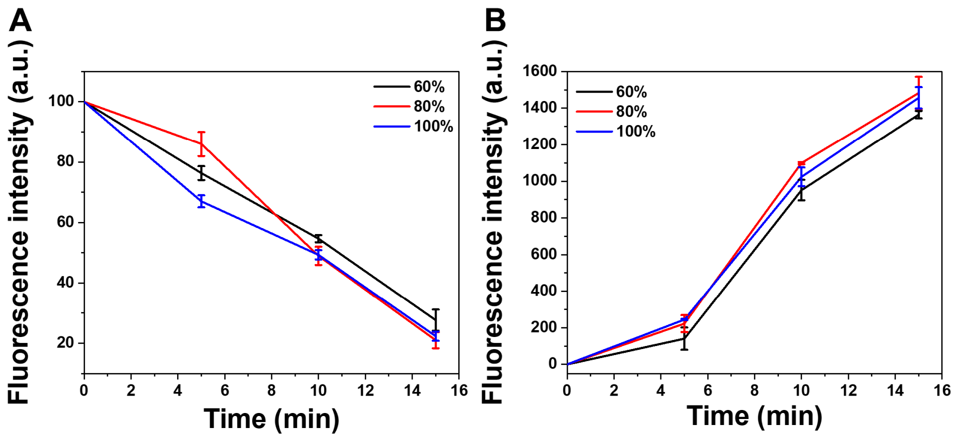

Figure S6. The generation rates of O_2 (A) and CO (B) at 60%, 80% and 100% ultrasonic intensities of POS-UCNPs NSs.

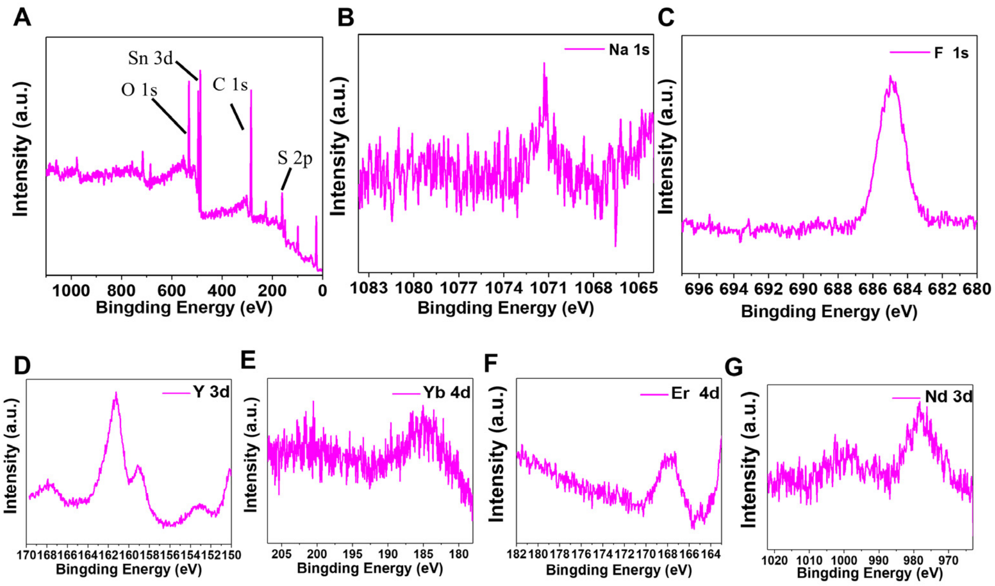

Figure S7. (A) XPS spectra of POS-UCNPs/ICG. (B-G) were a partial enlargement of Fig. A.

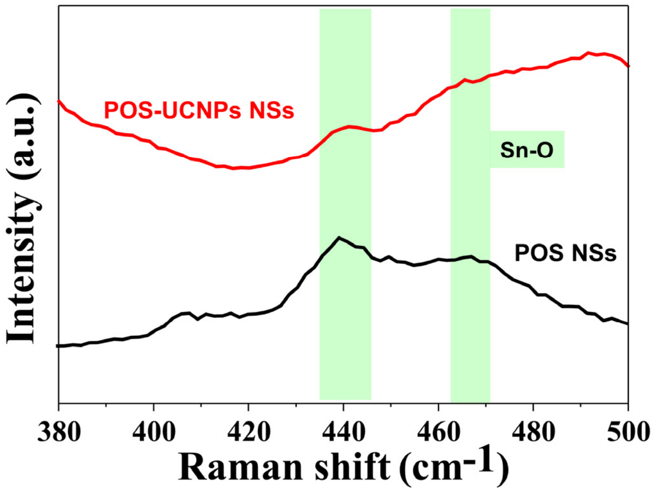

Figure S8. Enlarged Raman spectrum of POS NSs and POS-UCNPs NSs ranging from 380 cm^{-1} to 500 cm^{-1}.

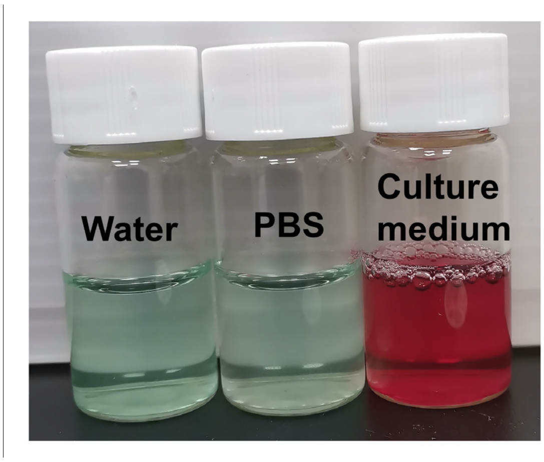

Figure S9. The picture of POS-UCNPs/ICG (100 μg·mL^{-1}) in deionized water, PBS and culture medium.

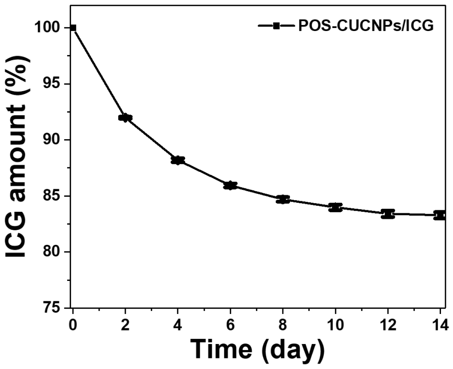

Figure S10. Water stability of POS-UCNPs/ICG over time.

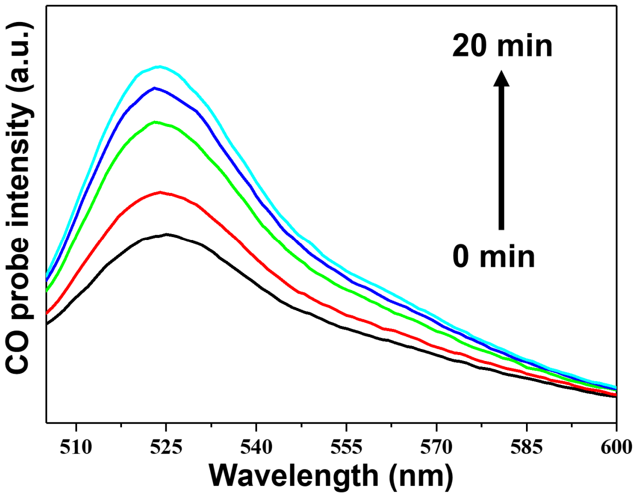

Figure S11. Fluorescence spectra changes of POS-UCNPs NSs solution together with CO probe and $PdCl_2$ upon 546 nm laser irradiation. The data were recorded every 5 min.

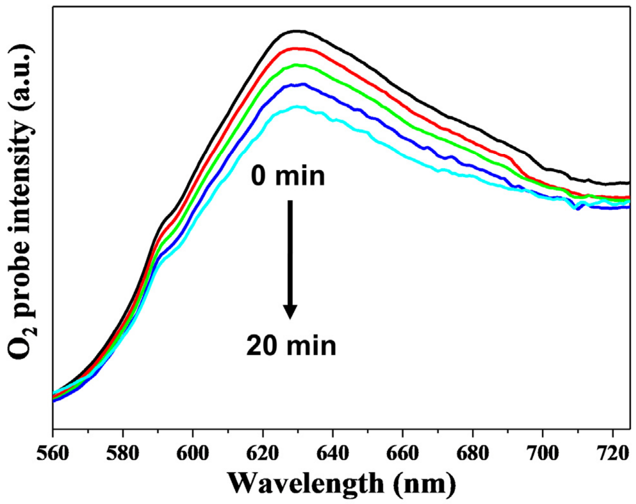

Figure S12. Fluorescence spectra changes of POS-UCNPs NSs solution with O_2 probe upon 546 nm laser irradiation. The data were recorded every 5 min.

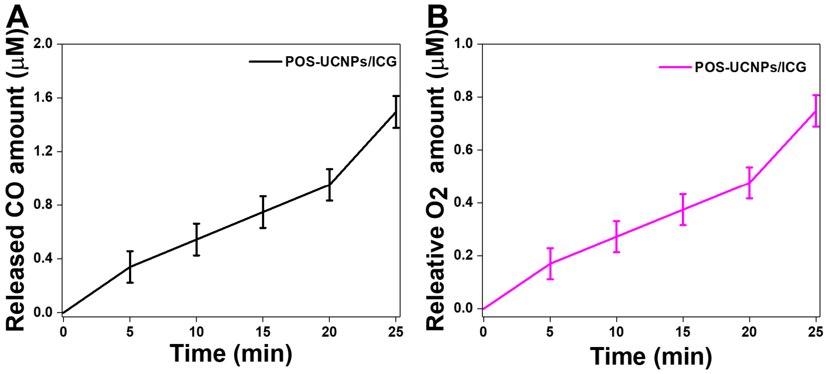

Figure S13. Quantitative detection of CO (A) and O_2 (B) under light condition.

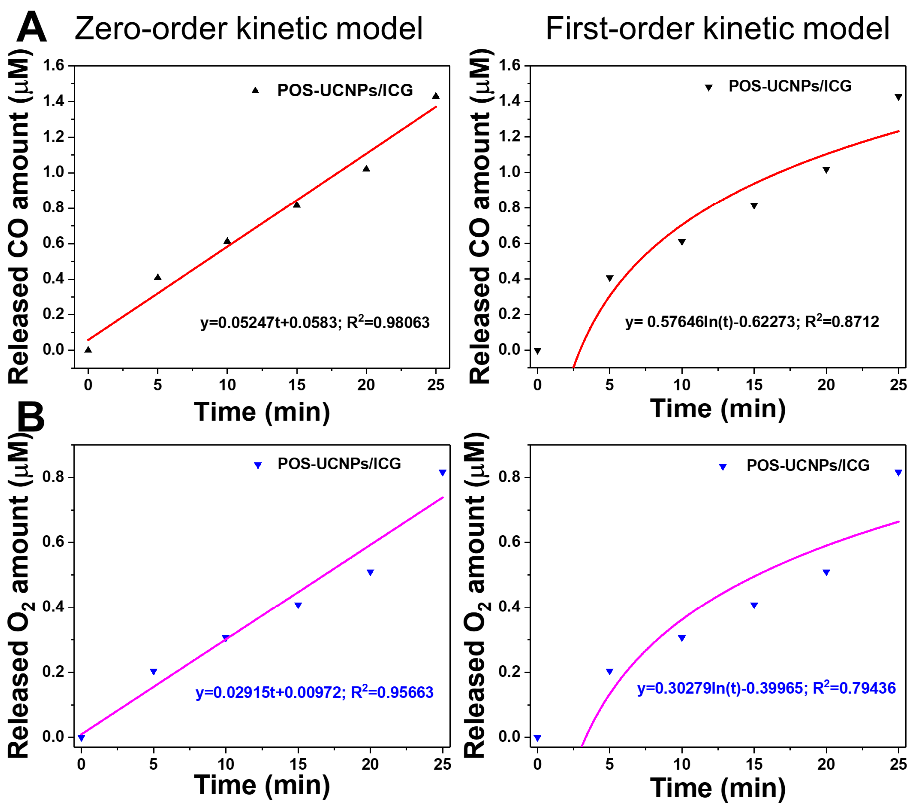

Figure S14. The Zero-order model and First-order model of CO (A) and O_2 (B) gas generation from NIR-irradiated POS-UCNPs/ICG.

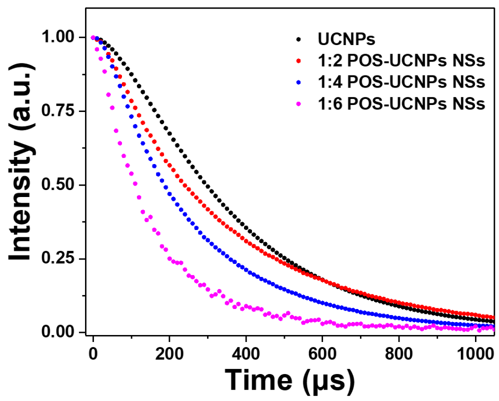

Figure S15. The lifetime of UCNPs and POS-UCNPs with different mass ratio.

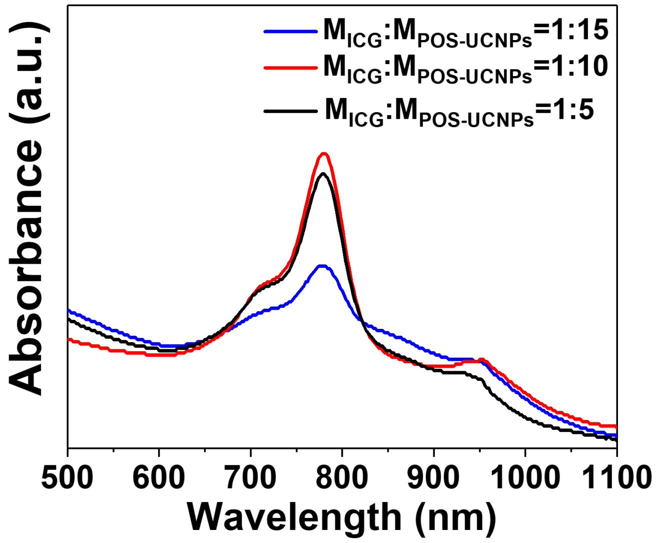

Figure S16. Absorption spectra of ICG and POS-UCNPs NSs with different mass ratios. The mass ratios of ICG: POS-UCNPs NSs are 1:15, 1:10 and 1:5, respectively.

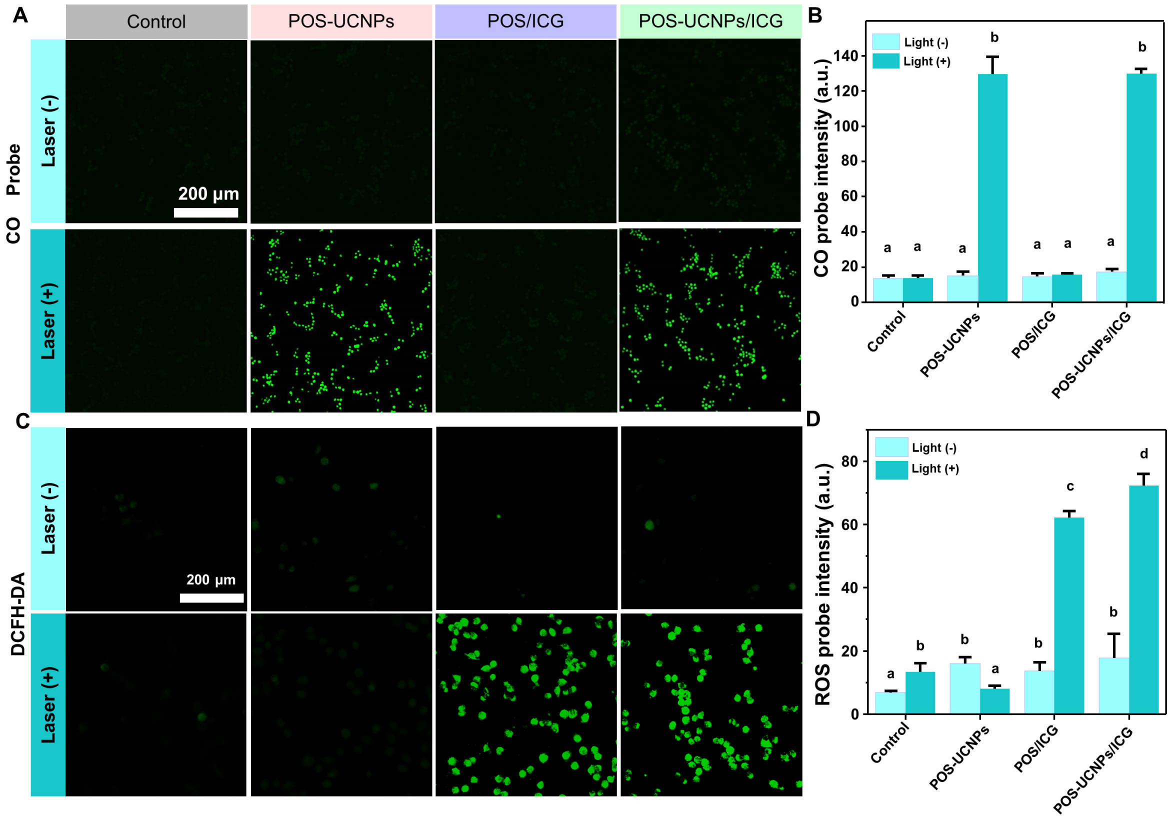

Figure S17. CO, ROS and cytotoxicity of POS-UCNPs/ICG at the cellular level. (A) CO probe staining images of macrophages after various treatments (PBS (control), POS-UCNPs, POS/ICG and POS-UCNPs/ICG). (B) The corresponding quantification for CO level by fluorescent intensity. (C) DCFH-DA staining images of L-929 fibroblasts after various treatments. (D) The quantitative analysis of ROS level by fluorescent intensity. Dissimilar letters represented statistical

differences between each other ($n = 6$, $p < 0.05$).

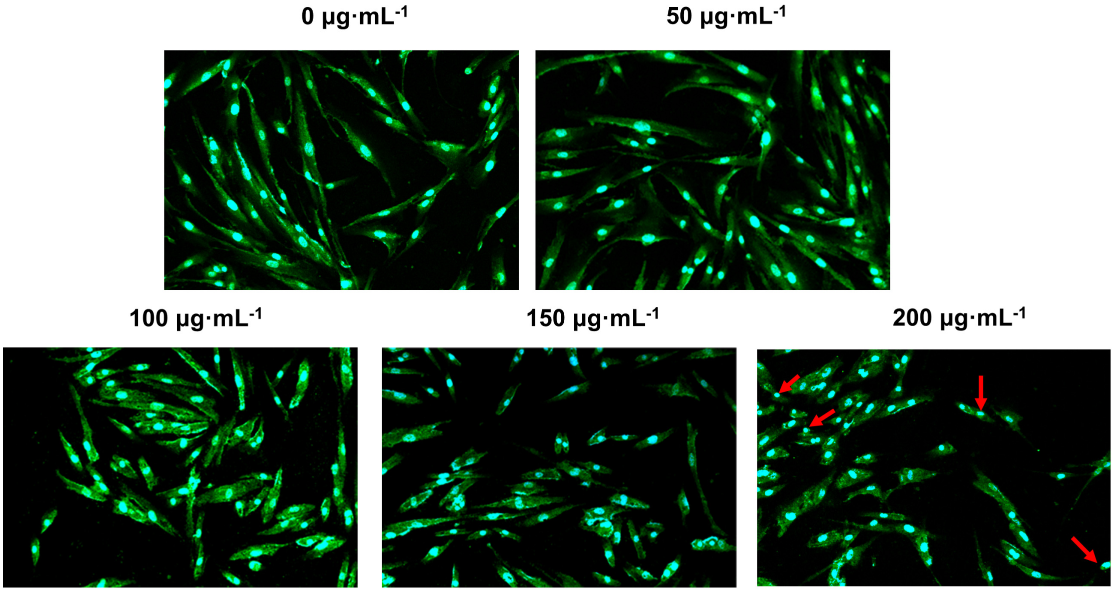

Figure S18. DAPI-FITC images of HGFs with different concentrations of POS-UCNPs/ICG.

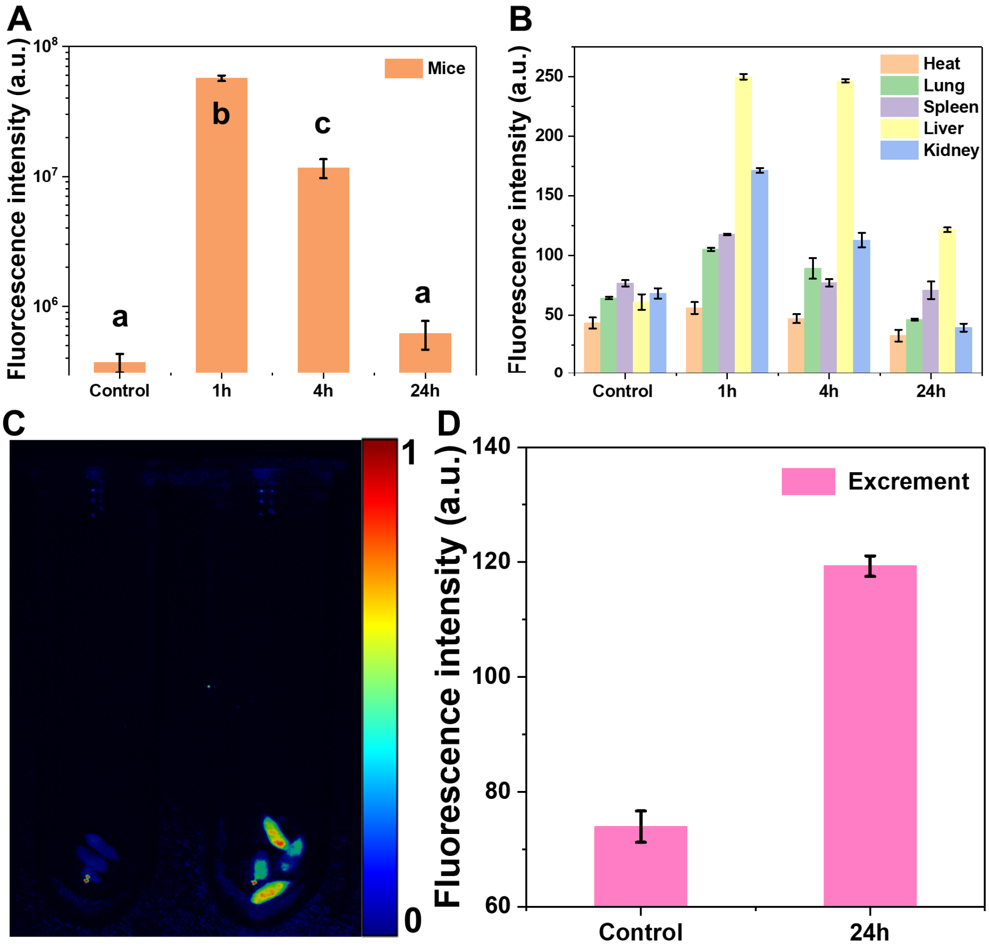

Figure S19. (A) The biodistribution of POS-UCNPs/ICG after injection in mice at different time points, semiquantitative fluorescent intensity in *vivo*. (B) The biodistribution of POS-UCNPs/ICG after injection in mice at different time points, semiquantitative fluorescent intensity of heart, lung, spleen, liver and kidney. (C) Fluorescent images of excrements of the mice treated with POS-UCNPs/ICG (right) and that of the control mice (left), respectively. (D) Semiquantitative fluorescent intensity of the excrements. Dissimilar letters represented statistical differences between each other ($n = 6$, $p < 0.05$).

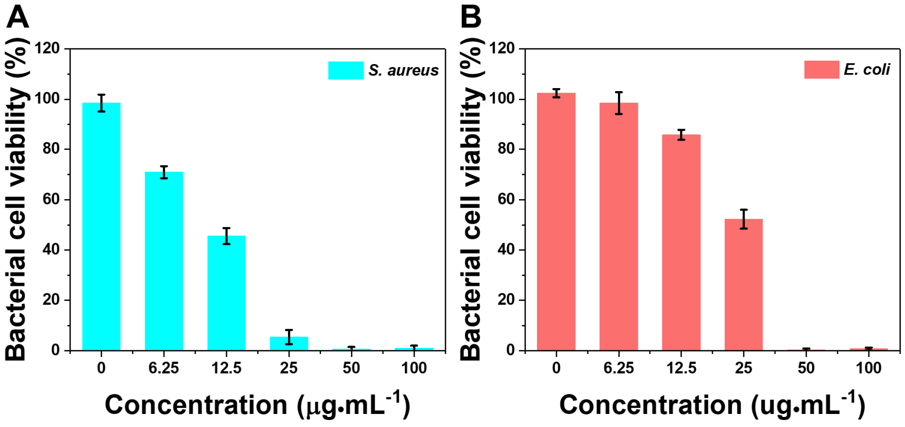

Figure S20. *S. aureus* (A) and *E. coli* (B) under a 808 nm laser (1 W·cm^{-2}) for 5 min at different concentrations of POS-UCNPs/ICG.

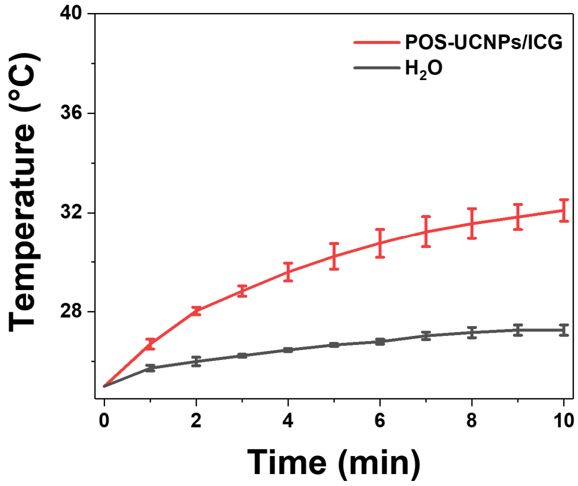

Figure S21. Temperature changes of POS-UCNPs/ICG solution (0 and 100 μg·mL^{-1}) with 808 nm irradiation (1 W·cm^{-2}).

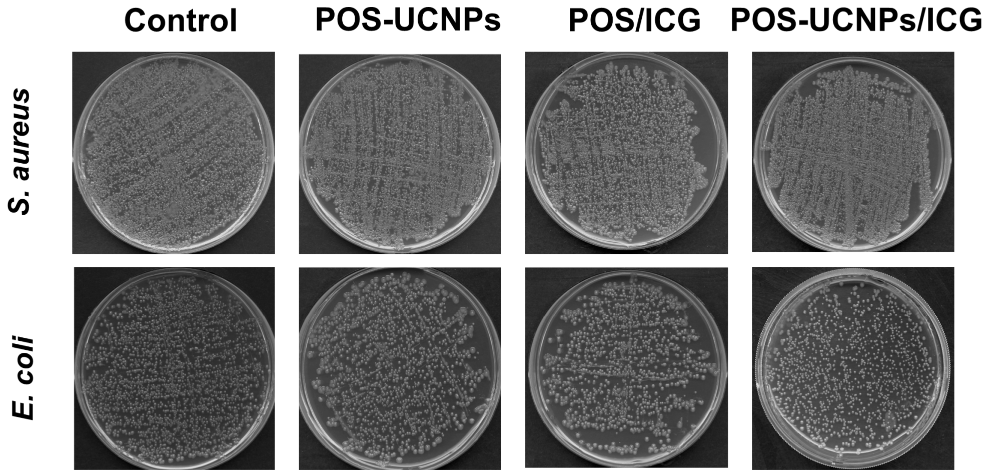

Figure S22. CFU images of *S. aureus* and *E.coli* after various treatments without light. Its diluting

factor was 10^5.

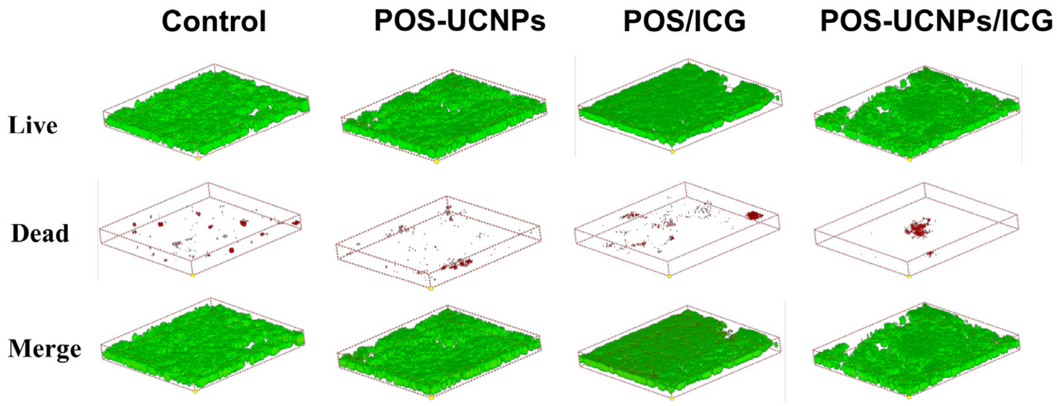

Figure S23. 3D dead/live images of *S. aureus* biofilms after various treatments without light.

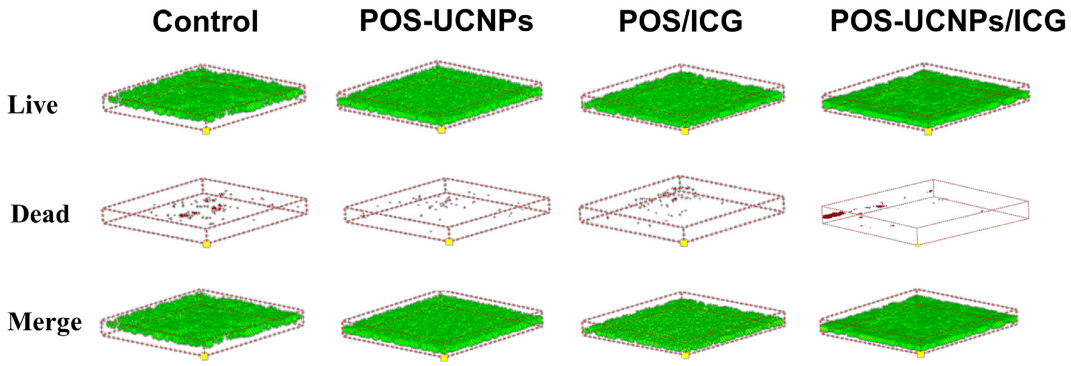

Figure S24. 3D dead/live images of *E. coli* biofilms after various treatments without light.

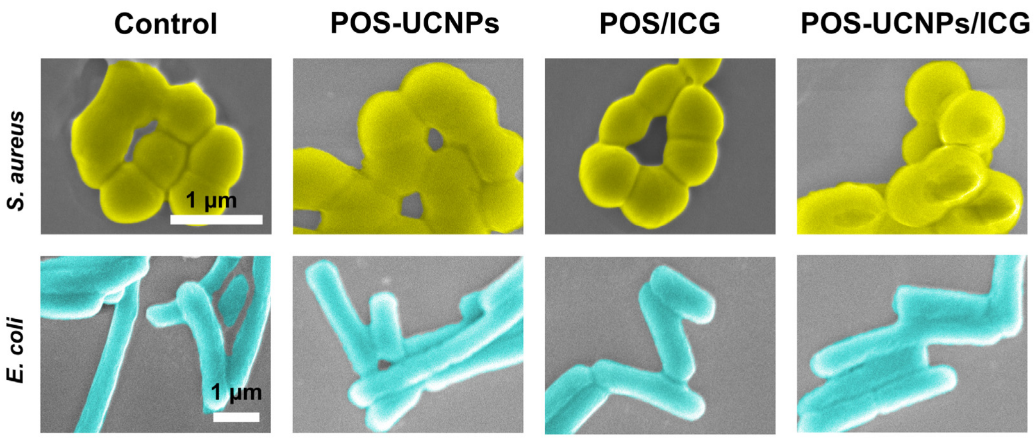

Figure S25. SEM images of *S. aureus* and *E. coli* after various treatments without light.

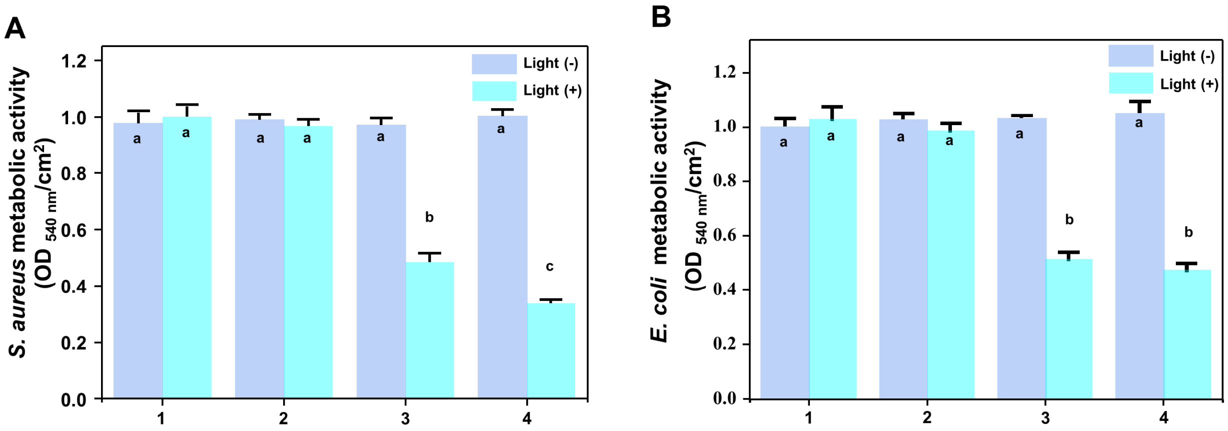

Figure S26. MTT assay for the metabolic activity of (A) *S. aureus* and (B) *E. coli* bioflms with different treatments (1: Control; 2: POS-UCNPs; 3: POS/ICG; 4: POS-UCNPs/ICG). Dissimilar letters represented statistical differences between each other (n = 6, $p < 0.05$).

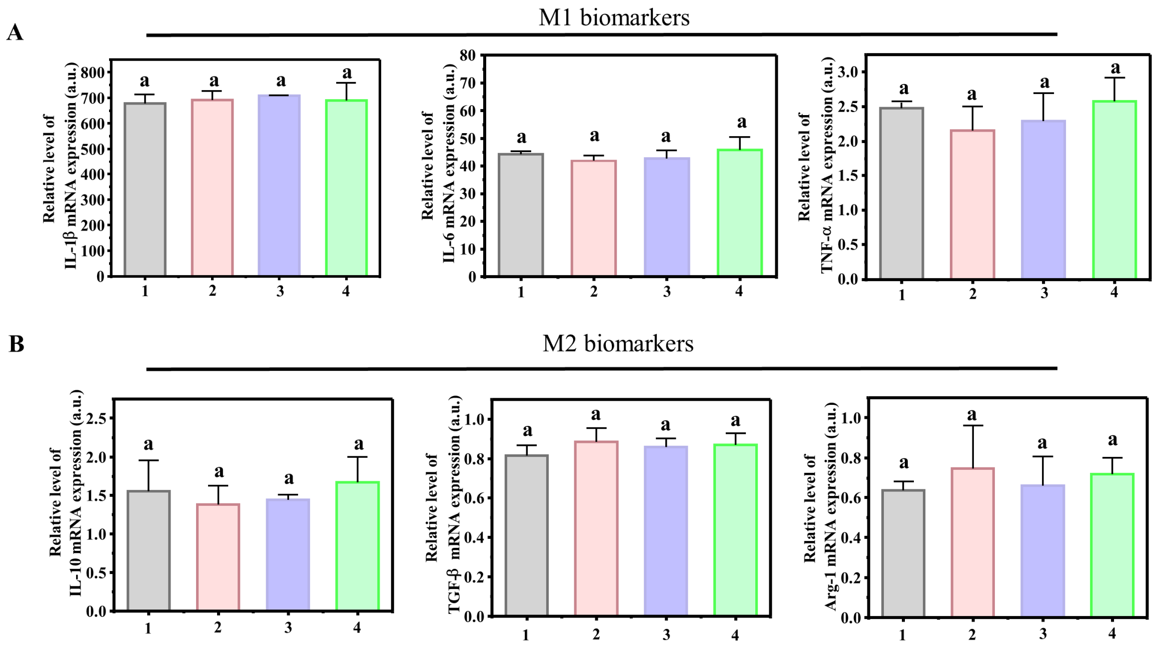

Figure S27. Gene expression of (A) M1 markers and (B) M2 markers after various treatments without light (1: Control; 2: POS-UCNPs; 3: POS/ICG; 4: POS-UCNPs/ICG). (The value for groups without stimulation equals to 1). Dissimilar letters represented statistical differences between each other (n = 6, $p < 0.05$).

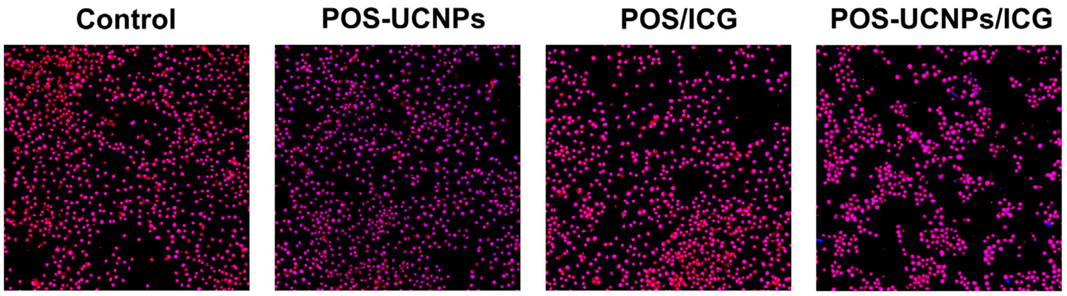

Figure S28. Immunofluorescent images of PI3K in macrophages after various treatments without light.

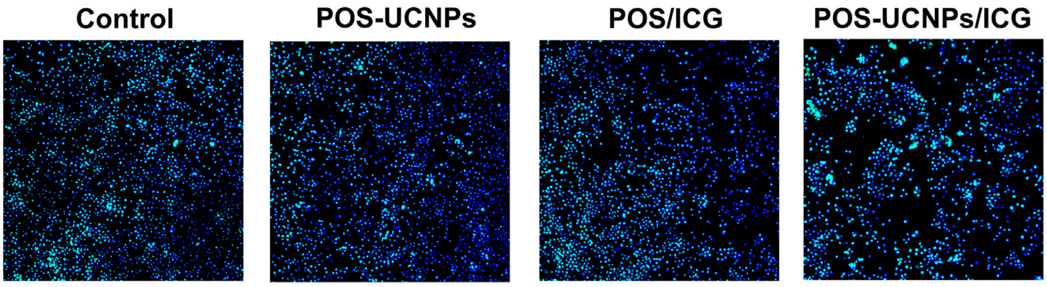

Figure S29. Immunofluorescent images of NF-kB in macrophages after various treatments without light.

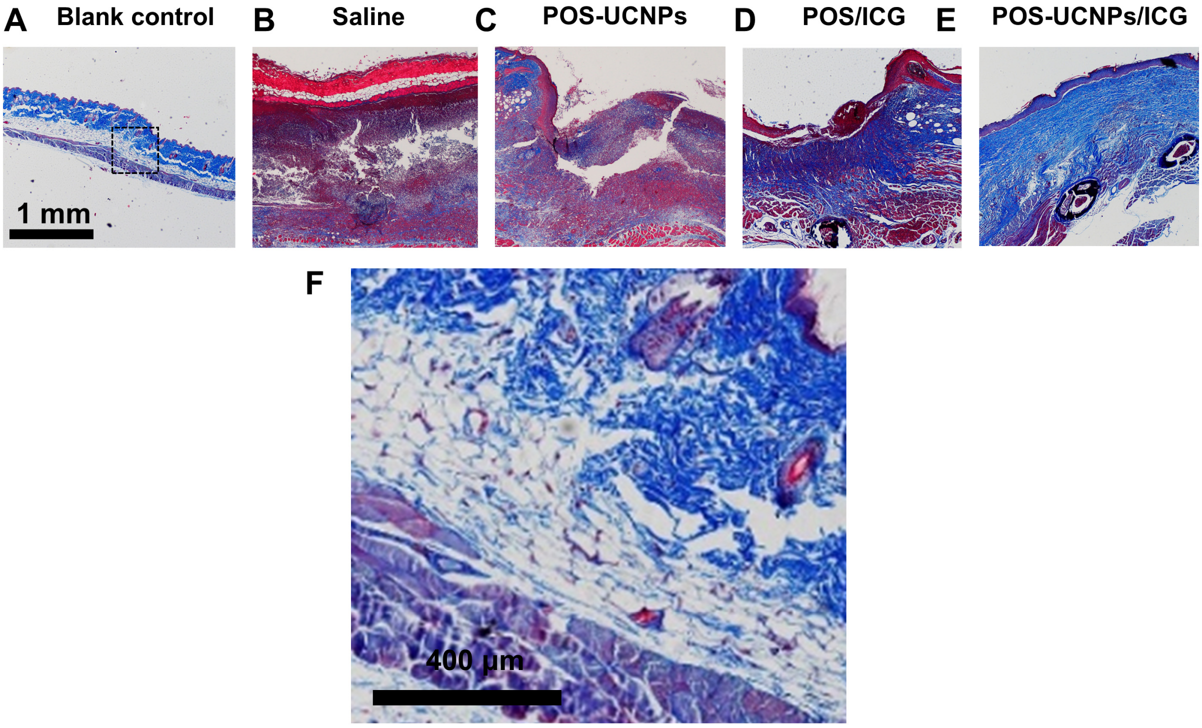

Figure S30. (A-F) Masson staining of various treated abscess tissues. (F) was a larger version of Figure (A).

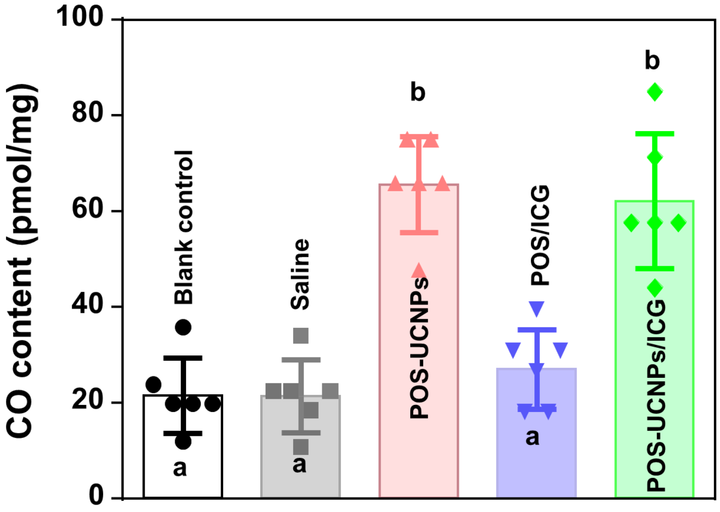

Figure S31. CO content in abscess site after various treatments. Dissimilar letters represented statistical differences between each other (n = 6, $p < 0.05$).

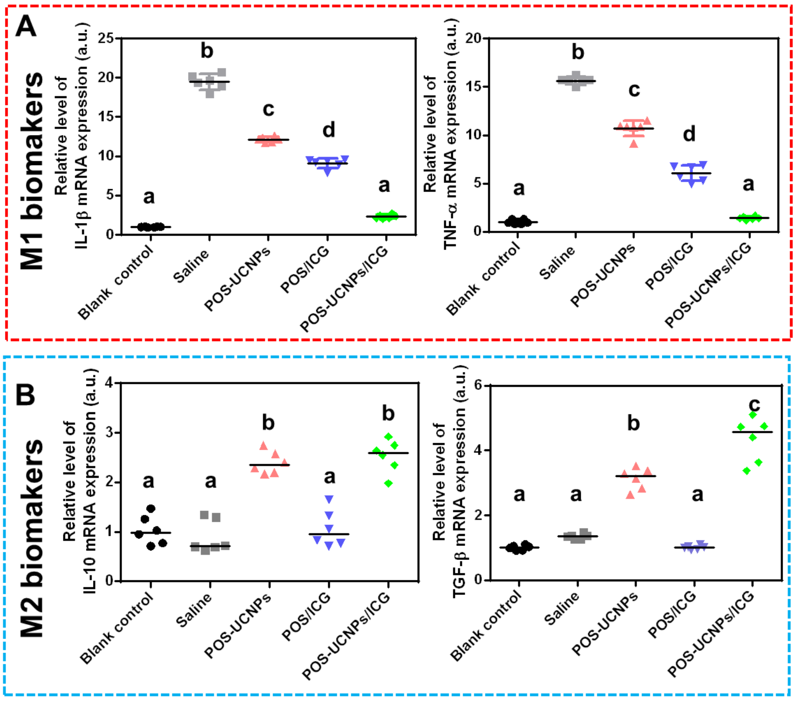

Figure S32. Quantitative analysis of mean mRNA expression of IL-1β and TNF-α in surrounding tissues of abscess sites in animal models. (B) Quantitative analysis of mean mRNA expression of TGF-β and IL-10 in surrounding tissues of infection sites in animal models (blank control equals to 1; Dissimilar letters represented statistical differences between each other (n = 6, $p < 0.05$).
